# Increased surface P2X4 receptors by mutant SOD1 proteins contribute to ALS pathogenesis in SOD1-G93A mice

**DOI:** 10.1007/s00018-022-04461-5

**Published:** 2022-07-19

**Authors:** Eléonore Bertin, Audrey Martinez, Anne Fayoux, Kevin Carvalho, Sara Carracedo, Pierre-Olivier Fernagut, Friedrich Koch-Nolte, David Blum, Sandrine S. Bertrand, Eric Boué-Grabot

**Affiliations:** 1 Univ. Bordeaux, CNRS, IMN, UMR 5293, 33000 Bordeaux, France; 2 Univ. Bordeaux, CNRS, EPHE, INCIA, UMR 5287, 33000 Bordeaux, France; 3 Univ. Lille, Inserm, CHU Lille, U1172, LilNCog, Lille, France; 4 “Alzheimer & Tauopathies”, LabEx DISTALZ, 59000 Lille, France; 5 Univ. Poitiers, INSERM, LNEC, UMR-S 1084, 86073 Poitiers, France; 6 Institute of Immunology, University Medical Center Hamburg-Eppendorf, 20246 Hamburg, Germany

**Keywords:** Purinergic signaling, P2X, TDP-43, Receptor trafficking, Spinal cord, Macrophage

## Abstract

Amyotrophic lateral sclerosis (ALS) is a fatal motoneuron (MN) disease characterized by protein misfolding and aggregation leading to cellular degeneration. So far neither biomarker, nor effective treatment has been found. ATP signaling and P2X4 receptors (P2X4) are upregulated in various neurodegenerative diseases. Here we show that several ALS-related misfolded proteins including mutants of SOD1 or TDP-43 lead to a significant increase in surface P2X4 receptor density and function in vitro. In addition, we demonstrate in the spinal the cord of SOD1-G93A (SOD1) mice that misfolded SOD1-G93A proteins directly interact with endocytic adaptor protein-2 (AP2); thus, acting as negative competitors for the interaction between AP2 and P2X4, impairing constitutive P2X4 endocytosis. The higher P2X4 surface density was particularly observed in peripheral macrophages of SOD1 mice before the onset and during the progression of ALS symptoms positioning P2X4 as a potential early biomarker for ALS. P2X4 expression was also upregulated in spinal microglia of SOD1 mice during ALS and affect microglial inflammatory responses. Importantly, we report using double transgenic SOD1 mice expressing internalization-defective P2X4mCherryIN knock-in gene or invalidated for the P2X4 gene that P2X4 is instrumental for motor symptoms, ALS progression and survival. This study highlights the role of P2X4 in the pathophysiology of ALS and thus its potential for the development of biomarkers and treatments. We also decipher the molecular mechanism by which misfolded proteins related to ALS impact P2X4 trafficking at early pathological stage in cells expressing-P2X4.

**Supplementary Information:**

The online version contains supplementary material available at 10.1007/s00018-022-04461-5.

## Background

ALS is the most common form of motor neuron (MN) disease, and is characterized by the selective loss of both motor cortical neurons and spinal MNs causing progressive paralysis, muscular wasting and death within 3–5 years after diagnosis. The etiology of ALS is unknown; however, the disease can be divided in two categories: 90% of ALS cases occur sporadically (SALS) while approximately 10% are familial (FALS)[1]. Originally, FALS have been shown to be linked with mutations in genes encoding Cu/Zn superoxide dismutase (SOD1), but have been more recently associated with TAR-DNA-binding protein 43 (TDP-43) [2], fused in sarcoma (FUS) [3] and predominantly C9ORF72 [4]. A pathological hallmark of ALS is the accumulation of proteinaceous inclusions in MNs and surrounding glial cells. Such protein inclusions are primarily composed of mutant SOD1 itself in FALS with SOD1 mutations. Interestingly, in SALS, while TDP-43 misconformation is largely observed [5], the presence of misfolded SOD1-containing inclusions was also revealed [6, 7]. ALS is thus considered as a protein misfolding disorder, and as such, is classified as a proteinopathy. ALS is also associated with inflammation in which glial cells in particular spinal microglia as well as peripheral macrophages participate to MN cell death and disease progression [1, 8–10]. Nevertheless, so far, neither biomarker, nor key target and an effective treatment against ALS has been found.

Purinergic signaling has been demonstrated to be involved in neurodegenerative diseases and recent data particularly suggest that ATP and P2X4 receptors could be attractive novel targets for understanding and fighting ALS disease [11–22]. In the central nervous system (CNS), ATP released by neurons and glial cells, participates to neuromodulation and neuroglial communication via P2 receptors including ionotropic P2X [23–26] and metabotropic P2Y receptors [27]. Alterations of purinergic signals have been associated with major CNS disorders including chronic pain, brain trauma, ischemia, epilepsy, multiple sclerosis (MS) as well as neurodegenerative diseases, such as Alzheimer disease (AD) or ALS [11–14, 28–33] with a pivotal role for P2X4 receptors (P2X4) [23–25, 34–38]. P2X4 is the main ATP-gated cation channel subtype expressed in neurons and glial cells in the CNS as well as in peripheral macrophages [23–25, 34–38]. In contrast to other P2X, P2X4 is constitutively and highly internalized and as a result, is essentially found in intracellular compartments, ensuring low surface expression in physiological condition. Intracellular P2X4 may promote vesicle fusion of endosomes or lysosomes [39, 40]. Intracellular P2X4 pools can also be rapidly mobilized and trafficked to the cell surface [41, 42]. In various pathological conditions, such as ischemia, chronic pain, MS and neurodegenerative disorders including ALS or AD, de novo P2X4 expression and/or increased surface P2X4 density by mobilization of intracellular pools were observed in microglia and/or in neurons [13, 14, 28, 30, 38, 43–46]. In the dorsal horn of the spinal cord, the specific upregulation of P2X4 in the microglia has been shown of paramount importance for the development of pain hypersensitivity caused by nerve injury [34, 43, 47]. Using a conditional internalization-defective P2X4mCherryIN knock-in mice, we recently showed that the increase in P2X4 at the surface of excitatory neurons reduces anxiety, impairs memory processing and alters synaptic plasticity in the hippocampus [48]. In the context of ALS, P2X4 have been shown to be upregulated in microglia [46] and the MNs of mutant SOD1-G93A (SOD1) mice before their death [30]. In addition, P2X4 exert a dual effect on MN survival depending on the ATP concentration [45] and ivermectin, a positive P2X4 modulator, has been shown to significantly extend the lifespan of mutant SOD1 mice [45]. Later work also reported that, parallel to neuronal degeneration, misfolded form of the mutant SOD1 protein was also detected by an antibody directed against the C-tail domain of P2X4 suggesting a more composite route for neurodegeneration behind P2X4 activation [49]. However, the role of P2X4 in ALS remains largely ill-defined.

In the present study, we demonstrate that misfolded mutant SOD1 proteins reduce the interaction between P2X4 and the protein responsible for its internalization, AP2 and thus lead to an increase in the surface density of P2X4 in cells expressing this purinergic receptor including peripheral macrophages at early ALS stages. In addition, we observe an upregulation of microglial P2X4 occurred during ALS progression. Finally, we report, using innovative double transgenic SOD1-G93A (SOD1) mice expressing non-internalized P2X4mCherryIN mice (namely P2X4KI) [48] or lacking the P2X4 gene (P2X4KO), that P2X4 contribute to ALS progression and survival in SOD1 mice.

## Methods

### Mice

hSOD1G93A mice (B6.Cg-Tg(hSOD1G93A)1Gur/J, background B6SJL) referred to as SOD1 mice, were obtained from The Jackson Laboratory (Bar harbor, ME, USA). Transgenic hemizygous SOD1 males were crossbred with females internalization-defective P2X4mCherryIN mice (P2X4KI, C57Bl6 background) to generate SOD1:P2X4KI mice (Fig. S2). General P2X4KI mice were obtained initially by breeding Floxed P2X4KI (flox/flox, expressing P2X4WT) with cytomegalovirus (CMV) promoter-Cre mice to substitute the internalization motif by the sequence of mCherry [48]. The ubiquitous activity of the CMV promotor induced the excision of the floxed cassette in the germinal cell lines and direct transmission to offspring. By breeding these animals, we thus obtained a constitutive P2X4KI mouse line (namely P2X4KI). Females invalidated for the P2X4 gene (constitutive P2X4KO mice, C57BL/6J background; [50]) were bred with SOD1 males to obtain SOD1:P2X4KO mice. SOD1 males mated with females homozygous Floxed P2X4KI provided SOD1:flox/flox offspring (namely SOD1:WT) were used as SOD1 mice and WT:flox/flox (namely WT:WT) used as controls. Second filial generation (F2) SOD1:P2X4KO and F3 SOD1:P2X4KI were used in this study and compared to F2 or F3 SOD1:WT or WT:WT mice, respectively. Genotypes were determined by polymerase chain reaction (PCR) on mouse tail DNA samples.

### Study approval

All experimental procedures complied with official European guidelines for the care and use of laboratory animals (Directive 2010/63/UE) and were approved by the ethical committee of Bordeaux. For SOD1 or double transgenic mice, clinical end stage was defined as the inability for the animal to right itself over a period of 20 s. For ethical reasons, animals were sacrificed at that point. Mice used in this study were between 5- and 17-weeks old males. For behavioral and survival studies, a minimum number of animals were kept until 20 weeks.

### Swimming test

Mice were placed in a 100 cm long, 6 cm wide swimming corridor filled with warm water (37 °C). The time to reach a platform placed at one extremity of the corridor was measured. Since physical activities can modify ALS progression, swimming test was conducted only every 10 days to avoid physical training.

### Motor scoring

To follow motor impairment all along ALS progression in mice, a motor scale was established based on the Fernagut et al. [51]. More precisely, five parameters were observed and rated on a scale of 0 to 2: (1) the general motor activity (exploration, rearing and grooming), (2) the postural adjustments of hind limbs while mice were suspended by the tail to monitor clasping (hind limbs retracted and touching the abdomen), (3) the increase in hind limb space (hind limb dystonia) associated with postural deficiency, (4) the presence of kyphosis, a characteristic curvature of the spine causes by the loss of dorsal muscular tone and (5) a postural challenge: the capacity of the mouse to resist to be tipped on the side and to get back on its feet.

### Immunohistofluorescence on mouse spinal cord

Mice were perfused intracardially with 0.9% NaCl and fixed with 2% PFA in 0.2 M phosphate buffer. Spinal cords were dissected, post-fixed 2–4 h in 2% PFA in 0.2 M phosphate buffer, washed and then transferred into 20% v/v glucose in PBS for 1 day. Spinal cord were then coated in cryogel and frozen in isopentane cooled to – 70 °C. Spinal cord floating cryosections (50 μm) were incubated in a blocking solution containing PBS, 0.05% Tween, 5% normal goat serum (NGS) for 45 min and then incubated with primary antibodies diluted in PBS 1% NGS overnight at room temperature: Rabbit anti-RFP 1:300; Rat monoclonal anti-mouse P2X4 antibody Nodu246 1:200; Mouse 1:1000; Chicken anti-GFAP 1:2000; Rabbit anti-Iba1 1:1000. Sections were then washed with PBS and primary antibodies revealed by incubation of brain slices in respective Alexa Fluor conjugated secondary antibodies for 1.5 h at RT. Sections were then washed with PBS and mounted in mounting medium containing DAPI. Images were taken using 49 Zeiss Imager M2 fluorescence microscope, Hamamatsu digital camera and Explora Nova MorphoStrider software with identical camera settings. Images presented in Fig. 4 were deconvoluted using Explora nova FluoD3 software. Contrast and luminosity were adjusted using Fiji software.

### RT-qPCR

Total mRNA was extracted from spinal cords and purified using the RNeasy Lipid Tissue Mini Kit (Qiagen). 1 µg of total RNA was reverse-transcribed using the High-Capacity cDNA reverse transcription kit with RNase inhibitor (Applied Biosystems). Quantitative real-time reverse transcriptase-PCR analysis was performed on Applied Biosystems™ StepOnePlus™ Real-Time PCR Systems using TaqMan™ Gene Expression Master Mix (Applied Biosystems). The thermal cycler conditions were as follows: 95 °C for 10 min, then 40 cycles at 95 °C for 15 s and 60 °C for 1 min. Reference of Taqman probes used are given in the Table S1. Cyclophilin A (PPIA) was used as internal control. Amplifications were carried out in duplicate and the relative expression of target genes was determined by the ΔΔCT method.

### *Xenopus laevis* oocytes electrophysiology

Oocytes were surgically removed from anesthetized Xenopus laevis and isolated as previously described [52, 53]. After nuclear co-injection of cDNAs encoding mouse P2X4WT and WT or mutated human SOD1 (G93A, G85R or G37R), oocytes were incubated in Barth’s solution containing 1.8 mM CaCl_2_ and gentamycin (10 mg/ml) at 19 °C for 1–3 days before electrophysiological recordings and/or biochemistry experiments. Two-electrode voltage-clamp recordings were performed as previously described [25, 54]. Briefly, recordings were carried out at room temperature using glass pipettes (1–2 MΩ) filled with 3 M KCl solution to ensure reliable holding potentials. Oocytes were voltage clamped at -60 mV and membrane currents were recorded with an OC-725B amplifier (Warner Instruments) and digitized at 1 kHz on an Apple computer using Axograph X. Oocytes were perfused at a flow rate of 10–12 ml/min with Ringer solution, pH 7.4 containing in mM: 115 NaCl, 3 NaOH, 2 KCl, 1.8 CaCl_2_ and 10 HEPES. 100 μM of ATP was applied using a computer-driven valve system (Ala Scientific).

### Isolation of mouse peritoneal macrophages

Mice were deeply anesthetized with a mixture of ketamine (100 mg/kg) and xylazine (20 mg/kg) and peritoneal cells were collected by washing the peritoneal cavity with 2 ml of phosphate buffer saline (PBS) as described [55]. The suspension of peritoneal cells was centrifuged for 8 min at 2000 rpm at 4 °C and then immediately used for biotinylation experiments.

### Biotinylation assays

Surface biotinylation experiments were performed as described previously [23, 53, 54] from injected Xenopus oocytes and mouse peritoneal macrophages. Briefly, cells were incubated in an ice-cold Ringer (for oocytes) or PBS with calcium and magnesium containing 1 mg/ml sulfo-NHS-SS-biotin and incubated at 4 °C for 4 h (or overnight) under gentle agitation. Excess sulfo-NHS-SS-biotin was removed by washes with buffer and quenched by washes with ice-cold quenching buffer containing 100 mM of glycine.

### Co-IP and pull-down

For co-immunoprecipitation experiments, spinal cord proteins extracts (0.5 to 2 mg of proteins) were incubated overnight at 4 °C in the presence or absence of primary anti-AP2 antibodies (see Table S1) covalently immobilized to Antibody coupling resin (Co-IP kit, Pierce). Beads were washes four times and eluted with low pH buffer or SDS sample buffer and subjected to SDS-PAGE. Western blots were performed as described below with primary antibodies against SOD1 proteins.

For pull-down assay, biotinylated peptides were synthesized by Genscript (NJ). Peptide CT-X4 corresponds to the C-terminal sequence of the mouse wild type P2X4. As control peptides we used a mutated (CT-3A) peptide. In peptide 3A, AP2 binding domain residues YxxGL were replaced by three alanines (AxxAA). 100 µg of biotinylated peptides (10 µg/µL) were fixed on streptavidin resin during 3 h at 4 °C. After several washes in Tris Buffer Saline (TBS) 1X, binding 500 µg of total protein extract was performed overnight at 4 °C. Resin were washed with TBS before elution with 40 µL of Laemili 2X with 12% of β-mercapto-ethanol per sample. Half of the elution was used for western blotting. Pull down assay were compared with input (20 µg of total protein extract).

### Immunoblotting

Biotinylated cells or spinal cord tissues were lysed by sonication in homogenization buffer (10 mM HEPES, 0.3 M sucrose, pH 7.4) containing protease inhibitors. Then, the proteins were solubilized with 1% Triton X-100 under agitation at 4 °C for 2 h. After centrifugation at 10,000*g* for 15 min, the supernatants containing the total proteins were quantified using the BCA method. For biotinylation experiments, a fraction of the supernatant was kept to assess total receptor fraction (Vt). The remaining supernatant (Vs) was incubated overnight at 4 °C with Immunopure Immobilized Neutravidin to precipitate surface proteins. Beads (20 μl) were washed with homogenization buffer and eluted with one volume of SDS sample buffer. Total and surface proteins were separated on SDS-PAGE and revealed using either anti-P2X4 (1:1000), anti-RFP antibodies (1:1000), anti-AP2 (1:500), anti-actin (1:10,000), anti-SOD1 (1:1000), anti-GFAP (1:10,000) or anti-Iba1 (1:2000) and horseradish peroxidase (HRP)-conjugated secondary antibodies (1:5000). Western blots quantification was performed using Image J software. The surface/total ratio from each experiment was determined as described [48] using the following equation:

$$\frac{{{\text{Signal}} s \times \frac{Vt + Vs}{{Vs}}}}{{{\text{Signal }}S \times \frac{Vt + Vs}{{Vs}} + {\text{Signal }}T \times \frac{Vt + Vs}{{Vt}}}}.$$

### Statistical analysis

The number of independent experiments or animals (*n*), the statistical test used for comparison and the statistical significance (*p* values) are specified for each figure panel in the corresponding figure legend. *p* values of less than or equal to 0.05 were considered statistically significant. The data are presented as mean ± SEM. The data were analyzed and graphs were generated using GraphPad Prism.

## Results

### Mutant SOD1 proteins alter P2X4 internalization leading to increased number and function

**Fig. 1:**
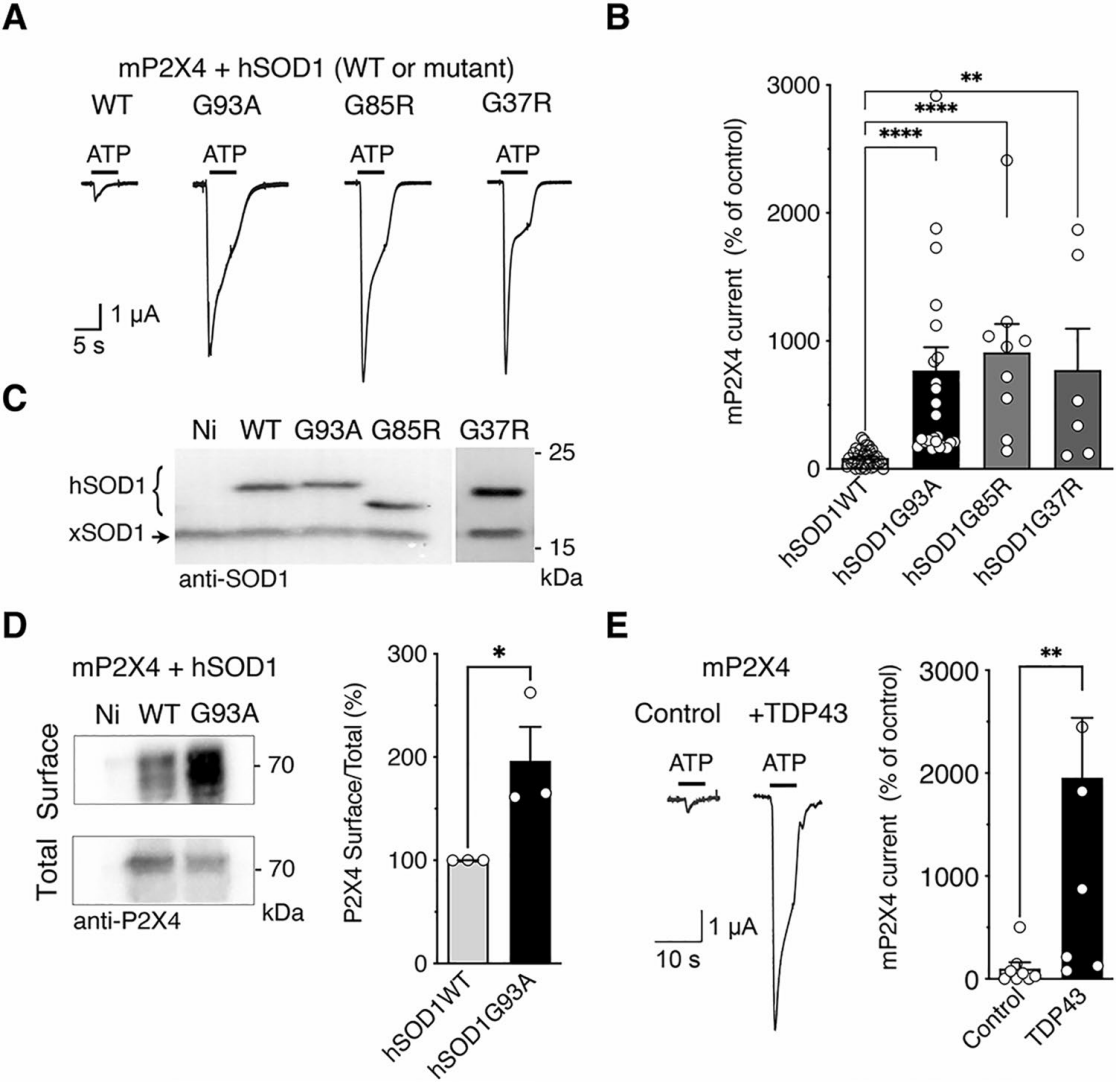
Mutant SOD1 proteins increase surface P2X4 number and function in vitro. **A** Representative currents evoked by 100 µM ATP in *Xenopus* oocytes co-injected with cDNAs encoding the murin (m)P2X4 and either the wild-type (WT) human SOD1 (hSOD1WT) or a mutant hSOD1 (hSOD1G93A, G85R or G37R). **B** Mean amplitudes of ATP induced-currents computed for all tested oocytes. ATP evoked P2X4 currents are strongly increased in cells expressing mutant hSOD1 (G93A, G85R and G37R) compared to those expressing hSOD1WT (** p < 0.01, ***p < 0.001, one-way ANOVA). **C** Western blot using anti-SOD1 antibodies confirmed the expression of the WT or mutated hSOD1 protein in addition to the endogenous Xenopus SOD1 (xSOD1) present also in non-injected oocytes (Ni). **D** Left, western blotting of surface and total proteins purified after protein surface biotinylation using anti-P2X4 antibodies from oocytes co-expressing mP2X4 and hSOD1WT or hSOD-G93A and non-injected oocytes (Ni). Right, normalized surface/total ratio shows that surface density of mP2X4 is increased in cells expressing SOD1G93A compared to those expressing hSOD1WT (*p < 0.05; unpaired *t* test). **E** Representative currents evoked by the application of 100 µM ATP in *Xenopus* oocytes co-expressing mP2X4 and human TDP43. Mean amplitudes of ATP induced currents computed for all tested oocytes (**p < 0.01; unpaired *t* test). Values of each cell or independent experiment are indicated on the graphs

P2X4 are highly and constitutively internalized by a clathrin-dependent endocytosis that involves an interaction between the endocytic adaptor protein-2 (AP2) and a non-classical binding motif (YxxGL), present exclusively in the C-tail of P2X4 [56–58]. Misfolding of mutant SOD1-G93A proteins revealed some sequence homology with the C-tail of P2X4. Misfolded SOD1 proteins resembling to the intracellular domain of P2X4 are indeed detected by anti-P2X4 antibodies directed against the C-tail of P2X4 [49]. Our working hypothesis was that mutated SOD1 proteins may interfere with the constitutive internalization of P2X4 protein, leading to an increase in surface P2X4. To address this question, we first investigated the potential impact of mutated SOD1 proteins on ATP-evoked currents recorded from *Xenopus* oocytes co-expressing mouse P2X4 (mP2X4) and either wild-type (WT) human SOD1 (hSOD1-WT) or mutant human SOD1-G93A (hSOD1-G93A) protein. Our results showed that the amplitudes of P2X4 currents evoked by 100 µM of ATP were significantly higher in cells expressing hSOD1-G93A compared to oocytes co-expressing P2X4 with hSOD1-WT (Fig. 1A–C). In the presence of other mutants of the human SOD1 protein involved in ALS, such as G85R and G37R, a similar significant increase in ATP-induced currents was also observed when compared with the P2X4/ hSOD1-WT co-expression condition (Fig. 1B). The similar level of expression of each hSOD1 proteins was verified after recordings by western blotting using anti-SOD1 antibodies (Figs. 1C and S1). Determination of the surface/total ratio of P2X4 from biotinylated oocytes by western blot using anti-P2X4 antibodies revealed a significant increase in surface P2X4 in the presence of hSOD1-G93A as compared to hSOD1-WT (Fig. 1D). These results suggest that misfolded SOD1 proteins alter P2X4 surface trafficking, leading to an increase in the number of surface P2X4 and a subsequent enhanced P2X4 response to ATP. Finally, as showed in Fig. 1E, we recorded a similar increase in P2X4 current amplitudes from oocytes co-expressing mP2X4 and another ALS-related protein, TDP-43. These results suggest that impairment in surface P2X4 trafficking is not exclusive to the mutated SOD1 proteins but could also be observed in the presence other ALS-related misfolded proteins.

**Fig. 2:**
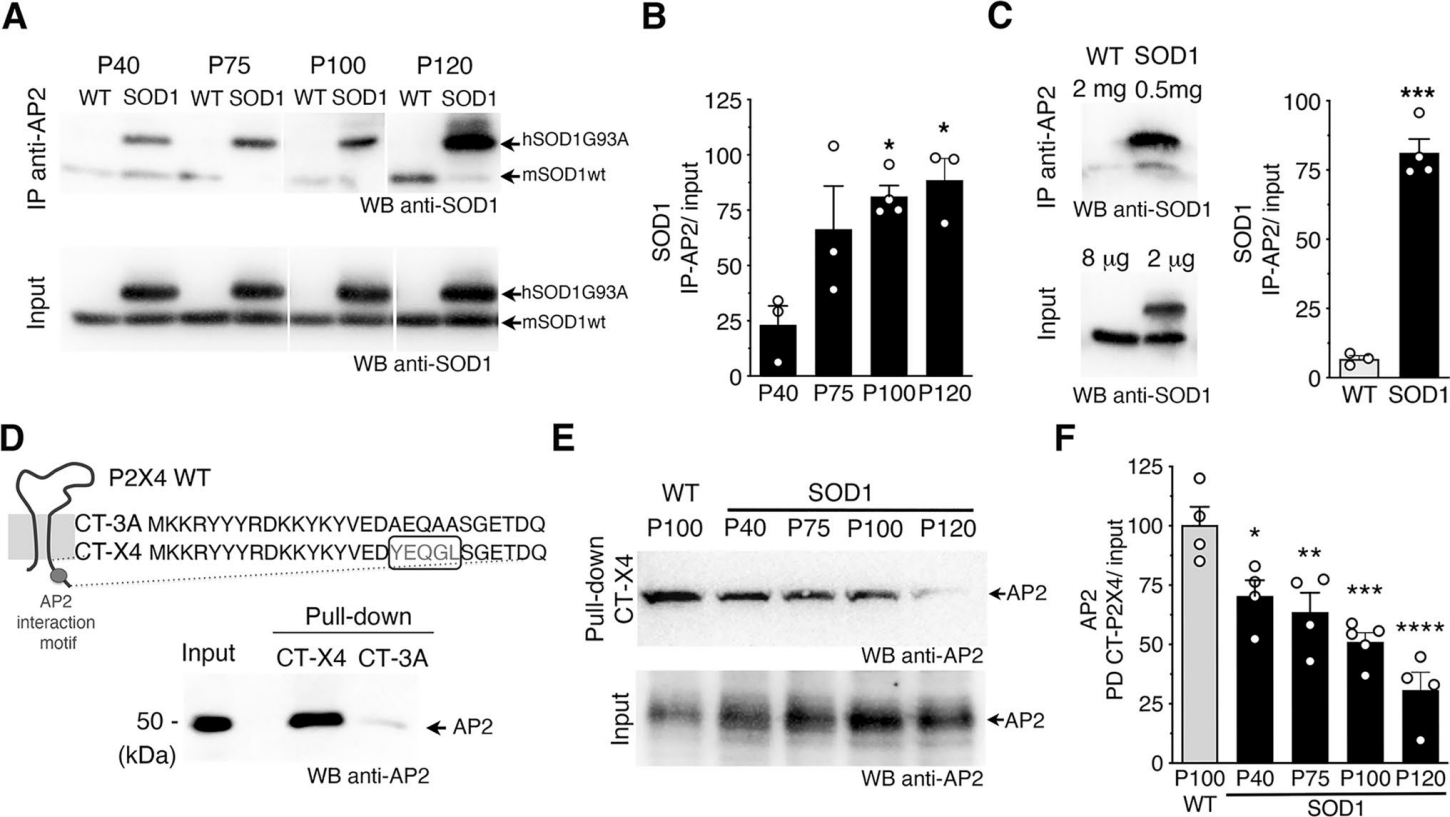
Mutant SOD1 proteins alter AP2 dependent endocytosis of P2X4 over ALS progression in the SOD1 mouse model. **A** Western blot analysis using anti-SOD1 antibodies after immunoprecipitation (IP) using anti-AP2 antibodies from spinal cord protein extracts of wild type (WT) and SOD1 mice at different stages (P40 to P120) revealed that SOD1-G93A co-immunoprecipitated with adaptor protein 2 (AP2) (see also panel E and Fig. S1B-C). Anti-SOD1 antibodies revealed in total proteins (input) one (mSOD1) or two bands (mSOD1 + hSOD1G93A) confirming the genotype of the mice tested. **B** The increase in SOD1 signals after IP over time suggests that the interaction between SOD1-G93A and AP2 increases during ALS pathogenesis (significantly different from P40, *p < 0.05, one-way ANOVA). **C** Co-IP control experiments performed with fourfold more proteins extracted from spinal cords of WT to reach similar amount of endogenous mSOD1WT and hSOD1G93A. SOD1 signals after IP with anti-AP2 show that solely SOD1G93A co-immunoprecipitated with AP2 (***p < 0.001, unpaired *t* test). **D** Pull-down assay using an immobilized peptide coding for the C-terminal domain of murin P2X4 (CT-X4) or a control peptide CT-3A (top) from spinal cord protein extracts of WT mice. After pull-down assay, eluted proteins were separated on a SDS-PAGE gel and revealed by western blot using anti-AP2 antibodies indicating that the C-tail of P2X4 interact specifically with AP2 while no signal was observed with CT-3A. **E** Pull-down assay using CT-X4 peptide of spinal proteins extracts from WT mice (P100) and SOD1 mice at P40, P75, P100 and P120 revealed using anti-AP2 antibodies as in D. Signals from inputs showed that AP2 expression is similar in all total protein extracts (see also Fig. S1 B–C). **F** The interaction between AP2 and P2X4 decreases over the time in SOD1 mice (significantly different from WT mice, in SOD1 mice at all ages (*p < 0.05, **p < 0.01, ***p < 0.001 one-way ANOVA)

The constitutive endocytosis of P2X4 is triggered by its interaction with AP2 [56–58]. We next sought to determine in a murine model of ALS, the SOD1G93A mice (hereafter referred to as SOD1 mice), whether SOD1-G93A proteins interact directly with AP2, and consequently act as negative competitors for the interaction between AP2 and P2X4. We thus performed immunoprecipitation (IP) experiments from spinal cord protein extracts from WT or SOD1 mice at different time points of the ALS progression (at 40 postnatal days (P40), P75, P100 and P120) using anti-AP2 antibodies (Fig. 2A, B). Following AP2 IP, western blot analysis using anti-SOD1 antibodies revealed a band corresponding to hSOD1G93A (and not to hSOD1-WT) indicating that SOD1-G93A co-immunoprecipitated with AP2 proteins. In addition, interaction between SOD1-G93A and AP2 significantly increased (*p* < 0.05, one-way ANOVA) over time, suggestive of a link with disease progression (Fig. 2A, B). To ensure the specificity of such interaction between mutant hSOD1-G93A and AP2, IP experiments were reproduced with larger quantities of total proteins extracted from spinal cords of WT mice to reach similar amount of endogenous mouse SOD1-WT than the one used for IP with hSOD1-G93A (Fig. 2C). In such conditions, solely SOD1-G93A co-immunoprecipitated significantly with AP2 while negligible signal was obtained for SOD1-WT (Fig. 2C). Furthermore, as shown in Fig. 2E and S1B-C, AP2 expression was similar and stable in WT and SOD1 mice at all stages tested, ruling out the possibility that changes in AP2 expression levels arise from changes in its interaction with SOD1-G93A.

Then, to determine whether interaction between SOD1-G93A and AP2 compete with the interaction between P2X4 and AP2, we conducted pull-down assays from spinal cord protein extracts from WT and SOD1-G93A mice at different time points of the ALS disease (P40, P75, P100 and P120) using, as a bait, an immobilized peptide corresponding to the C-terminal domain of mP2X4 (CT-X4) [52]. A control peptide (CT-3A) in which the internalization motif (Y^378^xxGL) was substituted by alanines (AxxAA) was used as negative control (Fig. 2D). Western blot using anti-AP2 antibodies showed that the C-terminal domain of P2X4 interacts specifically with AP2 proteins while the interaction is suppressed with the CT-3A peptide as bait in WT mice (Fig. 2D, E). Strikingly, as showed in Fig. 2E, AP2 signals decreased significantly after CT-X4 pull-down vs. input over time in SOD1 mice, supporting that the interaction between P2X4 and AP2 decreases with the progression of the disease. Together, our data demonstrate that increased interaction between mutant SOD1 proteins and AP2 parallels the reduced interaction between AP2 and P2X4. This alteration in P2X4 endocytosis is most likely responsible for the increased expression of P2X4 on the cell surface and thus to the enhanced response to ATP, measured in vitro and in ALS animal model [38].

### Internalization-defective P2X4KI mice reveal that increased surface P2X4 is beneficial for ALS pathogenesis in SOD1 mice

**Fig. 3:**
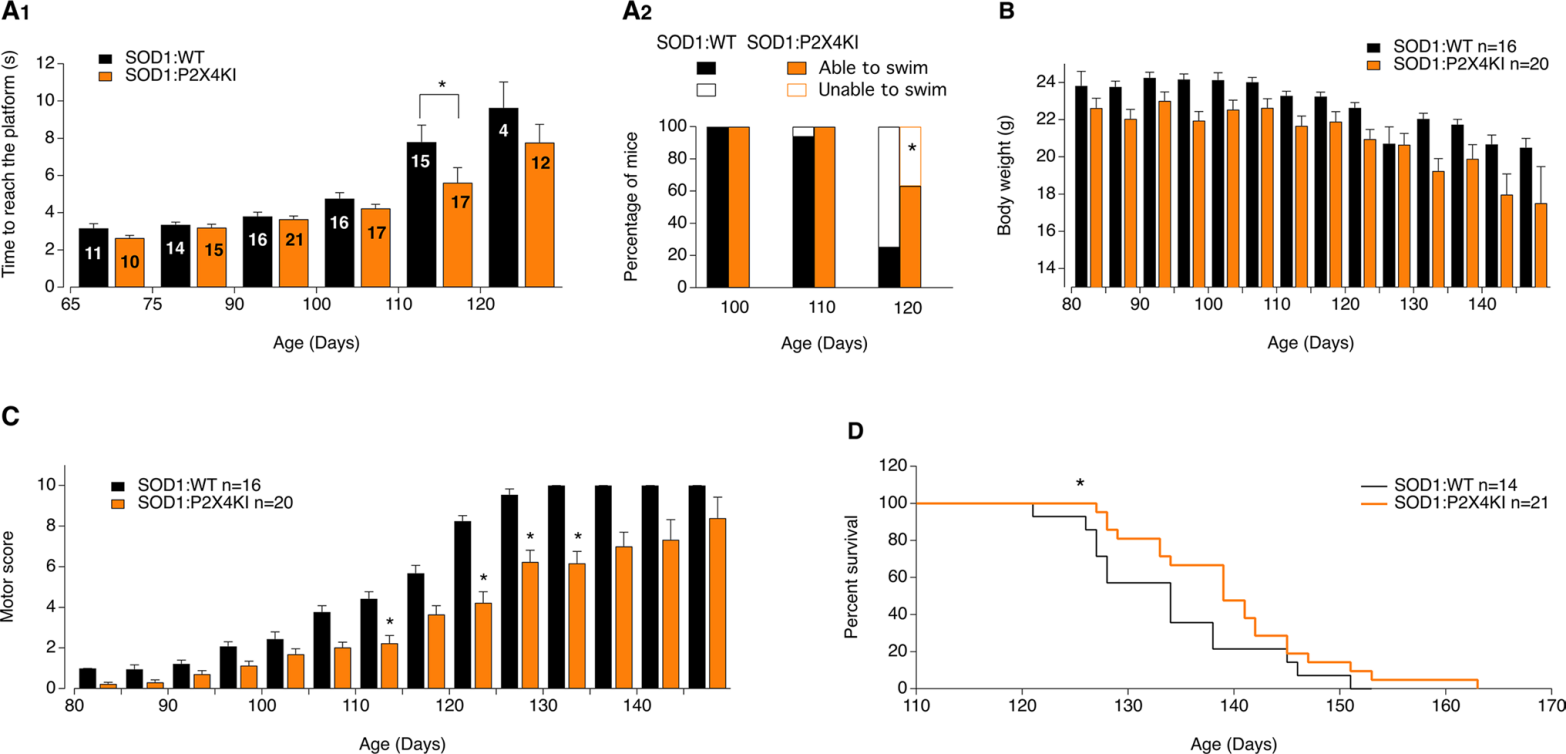
Increased surface P2X4 ameliorates ALS motor symptoms signs and life span in SOD1 internalization-defective P2X4KI mice. **A** Bar chart of the time to swim to a platform as a function of age (in days) for SOD1:WT (SOD1, black bars) and SOD1:P2X4KI (orange bars) mice (A1), (*p < 0.05; two-way ANOVA). Percentage of P100, P110 and P120 mice able or unable to swim in the two mouse lines tested (A2) (total = 16 SOD1 and 17 SOD1:P2X4KI for P100 and P110, chi-square, p = 0.3 at P110 and 16 SOD1 and 19 SOD1:P2X4KI P120mice, Chi-square, *p* = 0.02). **B**, **C** Bar charts of body weight (C) and motor score (D) as a function of age (in days) for SOD1:WT and SOD1:P2X4KI mice (*p < 0.05; two-way ANOVA). **D** Plot of percent survival for the two lines of mice tested (Gehan–Breslow–Wilcoxon test, *p* = 0.01). The data are presented as mean ± SEM. A number in bars indicate the number of animals tested

To determine to which extent increased surface expression of P2X4 contributes to ALS onset and progression, we crossed SOD1 transgenic mice (SOD1G93A) with constitutive internalization-defective P2X4mCherryIN knockin mice (P2X4KI) to obtain SOD1:P2X4KI (and littermate WT:P2X4KI) mice expressing enhanced surface levels of P2X4 [48]. Constitutive P2X4KI mice were obtained initially by breeding floxed P2X4KI (flox/flox, expressing P2X4WT) with the cytomegalovirus (CMV) promoter-Cre mice to substitute the internalization motif by the sequence of mCherry (see Methods and Fig. S2)*.* In parallel, we crossed SOD1 mice with control floxed P2X4KI to obtain SOD1:WT mice (and littermate WT:WT) of a genetic background similar to SOD1:P2X4KI mice and expressing native P2X4 (Figs. 3 and S2 and Table S1). In the SOD1-G93A mouse model, motor weakness is first detectable in the hind limbs. As adult mice swim exclusively with their hind legs [59], swimming appeared as a discriminatory test to evaluate the locomotor performances of the different mouse models. We therefore tested the time spent by mice to swim from one side of a corridor to a platform. In both SOD1:WT and SOD1:P2X4KI mice, we observed a progressive increase in the time to reach the platform with age with values reaching a significant difference from baseline in 110 days-old mice (P110) animals (Fig. 3A1). This time was however significantly reduced in SOD1:P2X4KI compared to SOD1:WT mice (Fig. 3A1) at P110, supporting transient motor improvement. We observed that the number of mice able to swim progressively decreased between P100 and P120. Although, all the mice tested were able to swim at P100 regardless of their genotype, some failed to perform the motor task at P110. At that stage, no significant difference was found between the two mouse lines tested (5 on 28 SOD1 and 1 on 24 SOD1:P2X4KI, Fig. 3A2, *p* > 0.05). In contrast, a significant difference was reached between SOD1 and SOD1:KI mice at P120 with only 25% (4 on 16 mice) of the SOD1:WT mice able to swim and 63% (12 on 19 mice) of age-matched SOD1:P2X4KI mice (Fig. 3A2, *p* < 0.05). The body weight loss observed during the disease progression in SOD1:WT mice was similar in SOD1:P2X4KI mice (Fig. 3B). To further evaluate the progression of the disease, we developed a motor scoring based on Fernagut and collaborators [51] to assess disease onset, progression and end-point for the different mouse genotypes. Increased surface P2X4 in SOD1:P2X4KI mice significantly slowed down the progression of motor symptoms as attested by the reduced motor score measured at several time points (Fig. 3C, *p* < 0.05). In accordance with the improved phenotype of SOD1:P2X4KI, the latter also exhibited a significant increased survival time as compared to SOD1:WT animals (Fig. 3D, *p* < 0.05). Because misfolded proteins in ALS proteinopathy increase surface P2X4 density, we originally reasoned that non-internalized P2X4KI would exacerbate ALS disease. In sharp contrast, our results show that pan-cellular increase of surface P2X4 delayed both motor signs and death in SOD1:P2X4KI mice.

### P2X4 expression switches from motoneurons to microglia within the spinal cord of SOD1 mice and parallels changes in astrocytic and microglial markers

**Fig. 4:**
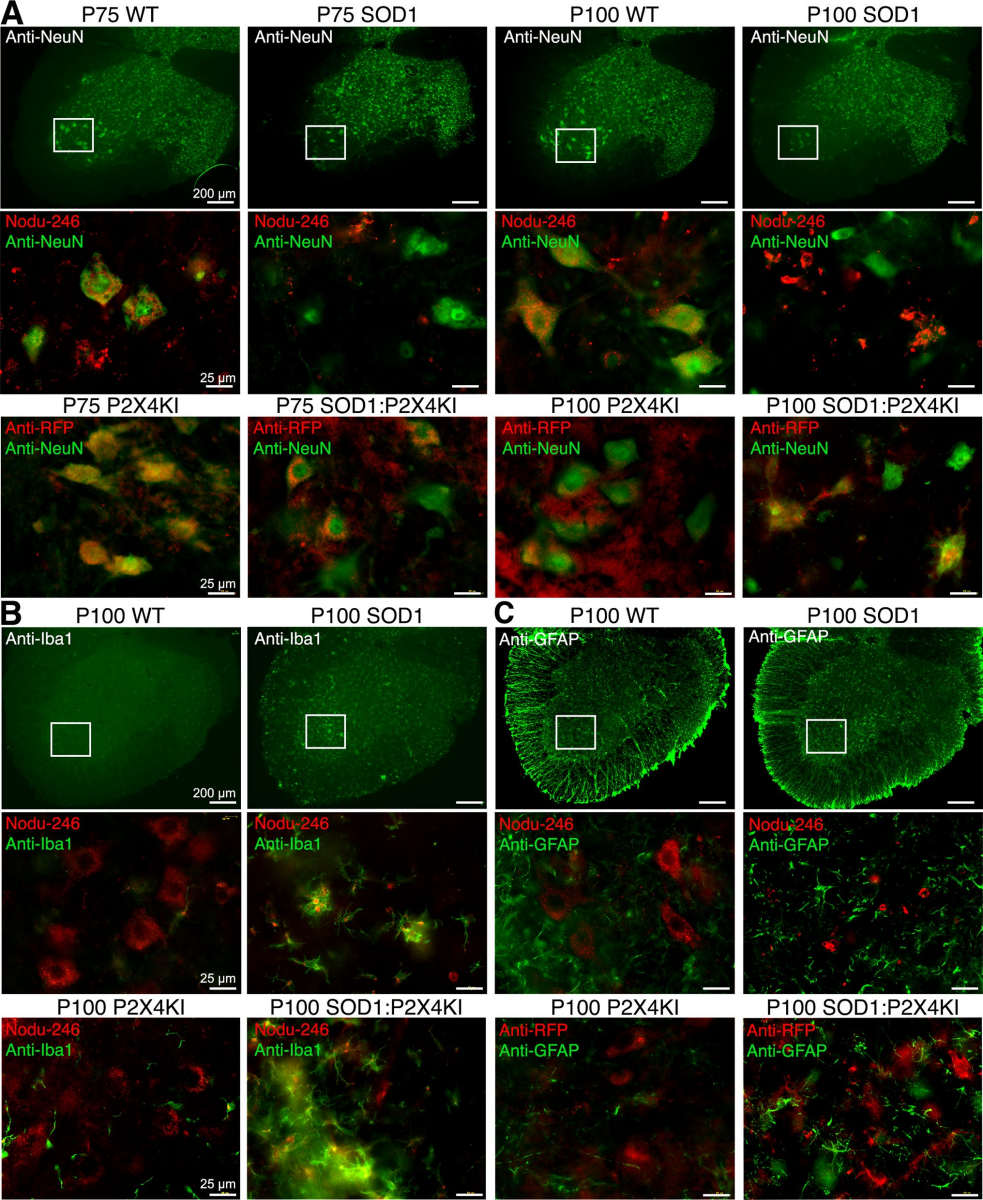
P2X4 expression switches over the time from motoneurons to microglia within the spinal cord of SOD1 mice. **A**–**C** P2X4 immunoreactivity in lumbar spinal cords from WT, SOD1:WT (SOD1) WT:P2X4KI (P2X4KI) and SOD1:P2X4KI mice at pre- (P75) and symptomatic (P100) phases of ALS. Expression of P2X4 in WT or SOD1 mice revealed with a rat monoclonal P2X4 antibodies (Nodu-246) and expression of P2X4KI revealed with anti-RFP in P2X4KI or SOD1:P2X4KI mice at P75 and P100 (see Fig. S6). Nodu-246, Beno-271 or anti-RFP were revealed with secondary antibodies coupled to Alexa-564 (red). Neurons A, microglia **B** or astrocytes **C** were identified using primary antibodies against NeuN, GFAP or Iba1, respectively, revealed with secondary antibodies coupled to Alexa-488 (green). White frames indicate magnified areas. A P2X4 or P2X4KI are expressed in spinal neurons located in the ventral horn at P75 in SOD1 or SOD1:P2X4KI mice and at P100 solely in WT mice. **B**, **C** At P100 in SOD1 or SOD1:P2X4KI mice, the increase in Iba1 (**B)** and GFAP (**C**) staining reveals the astro- and micro-gliosis within the spinal cord during ALS progression. At P100, P2X4 staining is localized in Iba1-positive cells of SOD1 or SOD1:P2X4KI spinal cords indicating the increase in P2X4 expression mainly in microglia during the symptomatic phase (See also Fig. S4 and S5)

Since anti-P2X4 antibodies directed against the C-tail of P2X4 subunits (Alomone labs) used in previous work [30, 49] were shown to recognize also misfolded SOD1 proteins [49], we re-examined P2X4 localization in the spinal cord of WT and SOD1 mice using the rat monoclonal antibody Nodu-246 recognizing specifically the extracellular domain of mouse P2X4 in its native conformation [48, 60]. We also took advantage of P2X4KI mice, to detect P2X4 using anti-red fluorescent protein (RFP) antibodies directed against the RFP mCherry fused to P2X4 in spinal cords of WT:P2X4KI and SOD1:P2X4KI mice (Figs. 4 and S3). Double immunostaining using Nodu-246 or anti-RFP antibodies with neuronal (NeuN) marker, showed that P2X4 expression is faint and essentially restricted to large neuronal cell body in the ventral horn of WT or WT:P2X4KI mice at P75 and P100 (Figs. 4A, B, S4 and S5) in agreement with the data obtained in the tdTomato *p2xr4* reporter mice expressing d-Tomato under the control of P2X4 promoter [61]. Some neuronal P2X4 expression was also observed in SOD1:WT and more clearly in SOD1:P2X4KI mice at P75 before the onset of ALS symptoms (Figs. 4A, B, S4 and S5). At later stage (P100) where MNs are dying, P2X4 staining increases in small cellular processes of the spinal cord of SOD1:WT or SOD1:P2X4KI mice when both astrocytic (GFAP) and microglial (Iba-1) markers were found upregulated (Figs. 4B, C and S6). GFAP-positive astrocytes mainly localized in the white matter of the spinal cord of WT mice (Figs. 4C and S6) increased drastically in the grey matter of the spinal ventral horn at P100 in SOD1:WT mice. Similarly, reactive Iba1-positive microglia increased in the ventral horn of SOD1 and SOD1:P2X4KI mice (Fig. 4B and S6). Both were consistent with the astro- and microgliosis classically described during ALS pathogenesis [62–71]. Notably, P2X4 staining using Nodu-246 or anti-RFP was found colocalized with Iba1 but not GFAP-positive cells, supporting that P2X4 expression particularly rises in microglia during the symptomatic phase in both SOD1:WT and SOD1:P2X4KI mice (Fig. 4B, C and S5).

### Surface P2X4 density increases in macrophages of SOD1 mice before the onset and during the progression of the disease

**Fig. 5:**
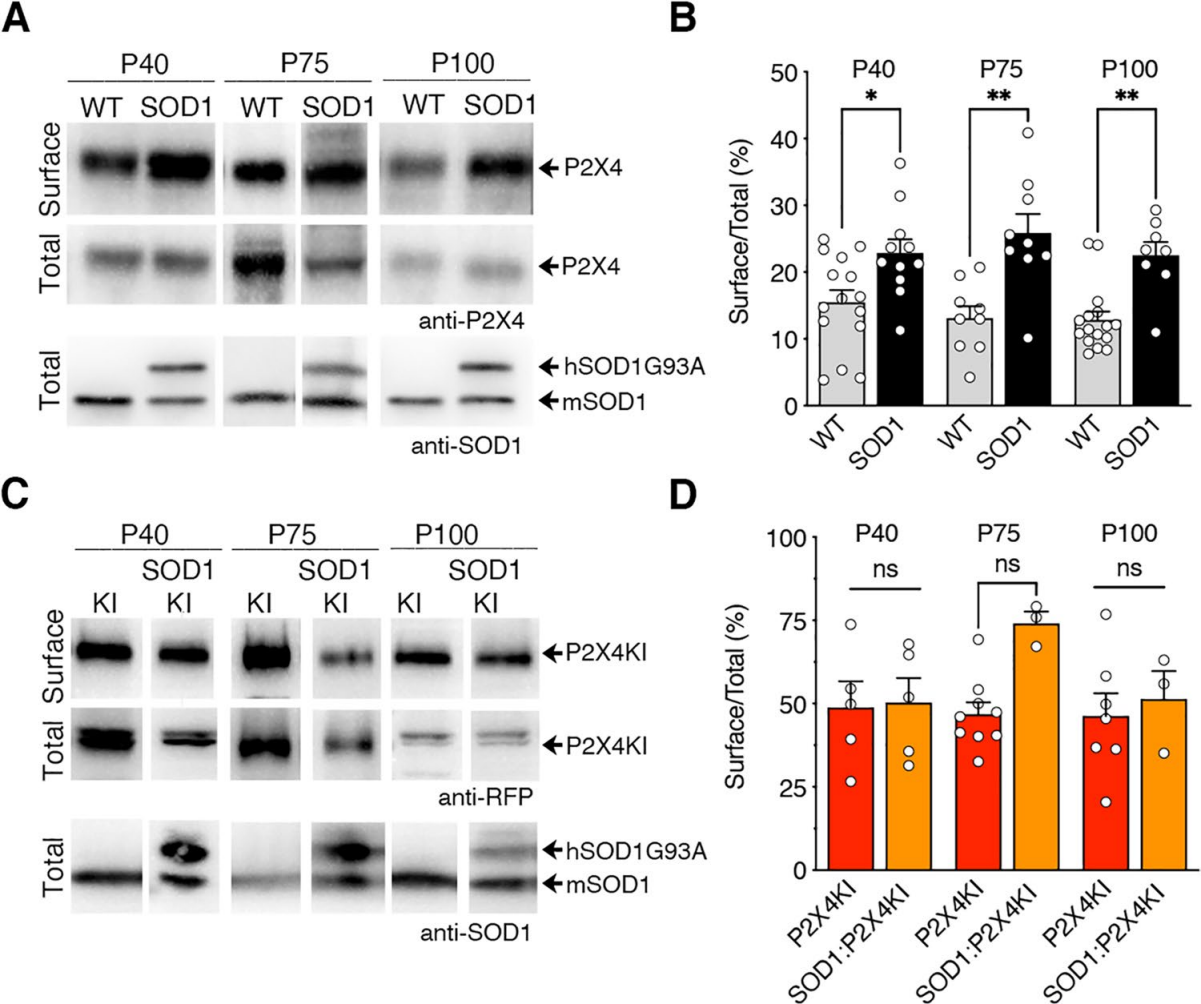
Surface P2X4 density is higher in macrophages of SOD1 as compared to WT mice before the onset and during the progression of the disease. **A** Western blotting of total and biotinylated surface proteins from peritoneal macrophages isolated from WT:WT (WT) and SOD1-G93A:WT (SOD1) mice at three time points (P40, P75 and P100). The anti-SOD1 antibody revealed 2 bands of different size corresponding to murine (m)SOD1 and human (h)SOD1-G93A confirming the genotype of the mouse. **B** Surface/total ratio shows that the number of surface P2X4 is increased in SOD1 macrophages as compared to WT at presymptomatic (P75) and symptomatic phase (P100). **C** Similar experiments from peritoneal macrophages isolated from WT:P2X4KI (P2X4KI) and SOD1:P2X4KI (SOD1KI) mice at the same 3 stages (P40, P75 and P100). **D** Surface/total ratio shows that the density of surface P2X4KI is similar between P2X4KI and SOD1:P2X4KI macrophages. (*p < 0.05, **p < 0.01, one-way ANOVA)

P2X4 is highly expressed by macrophages [34, 48, 72], belonging, as microglia, to the myeloid lineage. We recently showed that the low surface P2X4 density in macrophages from WT mice was significantly increased in P2X4KI mice by disruption of the constitutive internalization of P2X4mCherryIN [48] (Figs. 5 and S7). To determine whether misfolded SOD1 proteins induced an increase in surface P2X4 of macrophages during ALS, we performed biotinylation assays and western blot to measure P2X4 surface/total ratio from peritoneal macrophages of the different mouse lines used in this study at different time points (Figs. 5 and S7). Importantly, as shown in Fig. 5A, B, macrophages isolated from SOD1 mice exhibited a surface P2X4 density significantly higher than WT as soon as P40, largely before motor symptoms onset as well as at P75 and P100 (*p < 0.05; **p < 0.01). Moreover, the higher P2X4 surface density measured in macrophages from non-internalized P2X4KI mice was in contrast not significantly different than the one measured in SOD1:P2X4KI mice at all stages tested (P40, P75, P100) (Fig. 5C, D, and S7). The increase in surface P2X4WT trafficking measured in the presence of mutant SOD1 proteins as well as the fact that surface P2X4mCherryIN expression is unchanged in SOD1 mice are consistent with the results in Figs. 1 and 2 and strongly suggest that misfolded SOD1 proteins increase surface trafficking of P2X4WT by blocking its constitutive endocytosis in macrophages.

### Increase or absence of P2X4 transiently improves ALS and affects the microglial responses of SOD1 mice

**Fig. 6:**
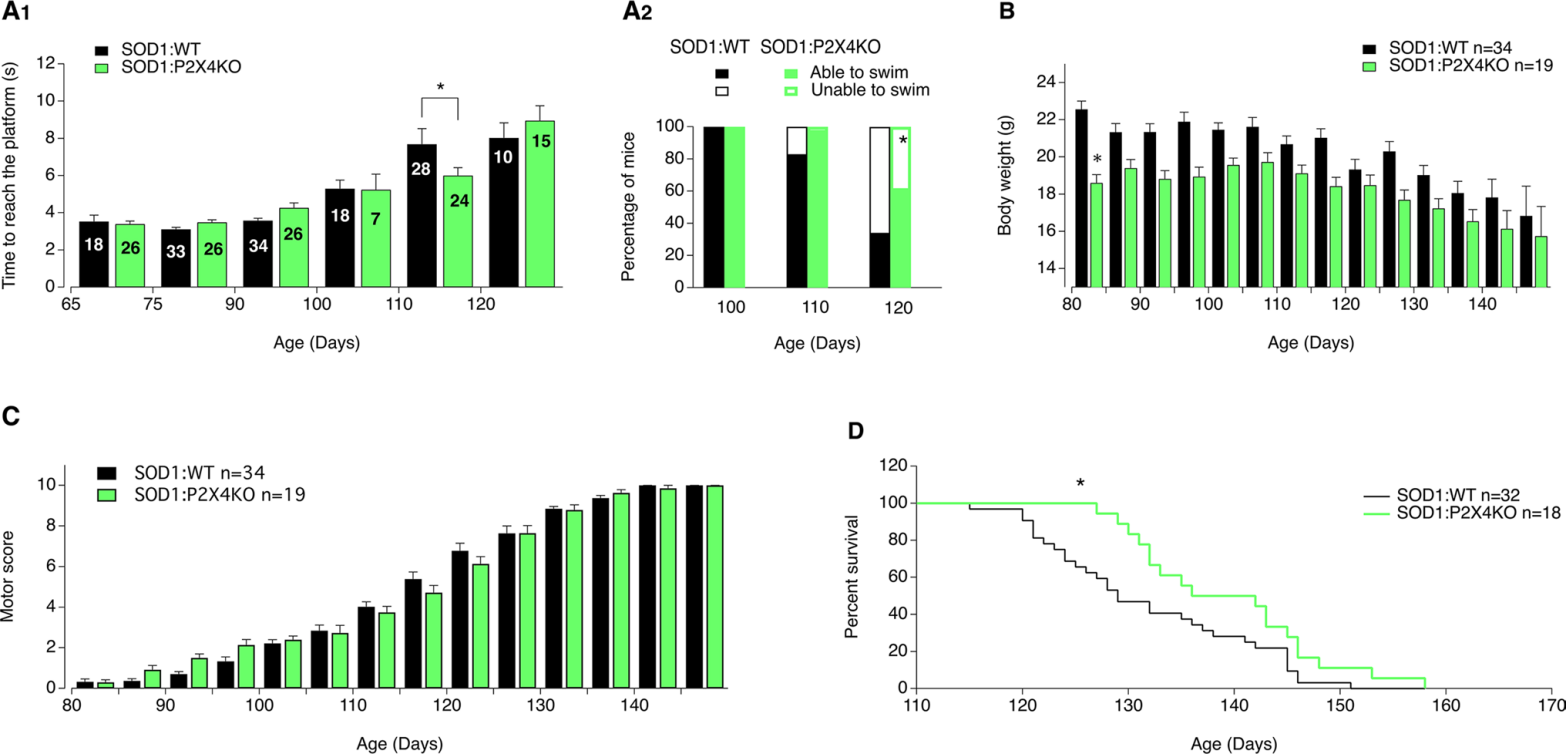
Absence of P2X4 ameliorates ALS motor symptoms and life survival in SOD1:P2X4KO mice. **A** Bar chart of the time to swim to reach a platform placed at one extremity of a corridor as a function of age (in days) for SOD1:WT (black bars) and SOD1:P2X4KO (green bars) mice (A1), (*p < 0.05; two-way ANOVA). Percentage of 100-day-old (P100), P110 and P120 mice able or unable to swim in the two mouse lines tested (A2) (total = 18 SOD1 and 17 SOD1:P2X4KO P100 mice; 33 SOD1 and 25 SOD1:P2X4KO P110 mice, Chi-square, *p* = 0.2 and 30 SOD1 and 25 SOD1:P2X4KO P120 mice, Chi-square, p = 0.04). **B**, **C** Bar charts of body weight (B) and motor score (C) as a function of age (in days) for SOD1:WT (black) and SOD1:P2X4KO (green) mice (*p < 0.05; two-way ANOVA). **D** Plot of percent survival for SOD1 (black) and SOD1:P2X4KO (green) mice (Gehan–Breslow–Wilcoxon test, *p* = 0.01)

Considering the benefit associated with enhanced cell surface expression of P2X4 in SOD1:P2X4KI mice (Fig. 3), we aimed at determining whether the loss of P2X4 would accelerate ALS pathogenesis. We thus compared motor performance and survival between SOD1:WT and SOD1:P2X4KO mice by reproducing the experiments described in Fig. 3. Surprisingly, the absence of P2X4 in SOD1:P2X4KO has also a significant positive impact on swimming performances (Fig. 6A) and survival as compared to SOD1:WT mice (Fig. 6A, D), although the magnitude of the effects was found lower than those observed with SOD1:P2X4KI mice. Body weight loss were similar between SOD1:WT and SOD1:P2X4KO as observed for SOD1:P2X4KI (Fig. 6B). In contrast to SOD1:P2X4KI mice (Fig. 3C), the loss of P2X4 did not impact the motor score of SOD1:WT (Fig. 6C). These observations strikingly support that the absence of P2X4 in SOD1:P2X4KO mice recapitulated, at least in part, the effects of increased surface P2X4 in SOD1:P2X4KI animals.

Finally, we examined the impact of either the absence or increased surface P2X4 on both glial activation and ALS-associated inflammation in the spinal cord of SOD1 mice [73–75]. We analyzed by western blot the Iba1 and GFAP protein levels and by RT-qPCR the expression of a panel of cytokines and mediators of the inflammatory response in the lumbar spinal cord in the different transgenic mouse lines at different times points (Fig. S8 and Fig. S9). Astrocytic GFAP, microglial P2Y12 markers and pro-inflammatory markers IL6, TNFα, IL1β as well as P2X4 and transcription factors IRF5 and IRF8 regulating P2X4 expression [76] increased significantly in SOD1 mice during the progression of the disease between presymptomatic (P40 or P75) and symptomatic stages (P100 and P120), as compared to WT mice. No significant change was observed for anti-inflammatory IL10 level between WT and SOD1 mice over the progression of the disease. Punctual changes can be observed for P2Y12, Iba1, TNFα or IL1-β between SOD1 and SOD1:P2X4KI or SOD1:P2X4KO mice (Fig. S8 and Fig. S9). The level of Iba1 as well as P2Y12, two microglial homeostatic markers, was found reduced in SOD1 P2X4 KO as compared to SOD1 mice at an early stage. At later stage of the disease, we observed a significant increase in the expression of IL1-β and P2Y12. These results suggest that P2X4 expression affects the microglial response in SOD1 mice and interestingly reveal that the effects differ depending on the state of the disease.

## Discussion

To date, we still miss biomarker and effective treatment to fight against ALS disease. Several studies highlighted the potential involvement of P2X4 in ALS, but its role remained elusive until now [30, 45, 46, 49]. In the present study, we demonstrated that mutant SOD1 proteins as well as TDP43 increase surface P2X4 density in cells expressing this purinoceptor in vitro*,* and that misfolded mutant SOD1-G93A proteins in SOD1 mice alter P2X4 endocytosis machinery before the onset of symptoms very likely leading to the increase in surface P2X4 expression observed in macrophages. In addition to be present in spinal MNs and macrophages, P2X4 expression increases in microglia through the activation of IRF8-IRF5 transcription factors during the symptomatic phase of ALS as commonly observed in neuropathic pain and neurodegenerative diseases associated with inflammation [32, 38, 76]. Moreover, we demonstrated that P2X4 is instrumental for ALS in SOD1 mice. Since misfolded proteins in ALS increase surface P2X4 density, we originally expected that increased surface P2X4 would exacerbate ALS disease in SOD1:P2X4KI whereas absence of P2X4 would ameliorate ALS in SOD1:P2X4KO mice. The most striking finding is that both increased surface P2X4 or P2X4 gene invalidation were able to transiently mitigate motor impairments and improve survival in SOD1 mice. Motor outcomes were measured using swimming test and general motor score. The swimming test may be considered more powerful since it is a functional readout that directly assesses a complex motor behavior requiring postural adjustments, limb coordination and strength. On the other hand, the motor score is less challenging addressing spontaneous parameters and postural challenge (see Methods). However, this motor score is nevertheless useful as a complementary behavioral readout to monitor the progression of the overall phenotype of the mice over time. Although both P2X4KO and P2X4KI led to a significant improvement of motor function of SOD1 mice as assessed using the swimming test, the motor score was able to disclose a positive effect on the progression of the motor disorder in P2X4KI mice, suggesting that the lack of P2X4 internalization had overall a stronger beneficial effect on the course of the disease.

The similar functional outcomes of both P2X4KO and P2X4KI mice is striking but several explanations can be proposed. P2X4 expressed in different cell types MNs, microglia or macrophage involved in ALS pathogenesis and neurodegeneration [9, 45, 46, 63, 64] may promote either beneficial or detrimental effects in specific cell types and/or at different stages of ALS progression since changes in the expression of microglial P2X4 (our results Fig. 4, S8 and [46]) and in ATP concentration were observed during ALS progression[45].

The dual action of P2X4 on MNs survival was previously reported and linked to changes in ATP concentration during ALS pathogenesis. Indeed, low ATP concentrations in combination with ivermectin, a specific positive modulator of P2X4, were shown to protect MNs from kainate induced excitotoxicity. In contrast, high ATP concentrations triggered deleterious effect on MNs [45]. Ivermectin is a positive allosteric modulator of P2X4 but it has been also proposed that ivermectin increases the number of surface P2X4 by modulating its trafficking [53, 77, 78]. Ivermectin administration at presymptomatic stage slightly extends (from 10%) the lifespan of SOD1 mice [45]. These data are therefore consistent with our observation that increased surface P2X4 density in SOD1:P2X4KI mice ameliorates ALS symptoms. This duality of P2X4 actions linked to ATP concentration in vitro could be transposed in vivo*.* Indeed, during the progression of the disease, ATP is massively released by dying cells and acts as a danger signal at symptomatic stage [79]. Similarly, a dual role of P2X7, that is mainly microglial, has also been reported in SOD1 models [80, 81]. Indeed, constitutive ablation of P2X7 in SOD1 mice is detrimental [80] whereas P2X7 antagonist administration specifically at symptomatic stage (P100), is beneficial [81]. These results support the existence of a time window during which a beneficial action of P2X4 activation in ALS is observed.

P2X4 are constitutively internalized and mainly retained intracellularly in endosomes and lysosomes. [42]. Intracellular P2X4 may form functional channels activated by luminal ATP and changes in pH, promoting vesicle fusion of endosomes or lysosomes [39, 40]. Intracellular P2X4 modulates lysosomal function such as phagocytic and autophagy pathways with positive or negative implications. Intracellular P2X4 improves the symptoms of experimental autoimmune encephalomyelitis (EAE) by promoting remyelination [32], but accelerates breast cancer progression [82]. The similar positive effects on ALS symptoms observed in both P2X4KO and non-internalized P2X4KI mice could be explained by a prominent and negative role of intracellular lysosomal P2X4 function in ALS [32]. Indeed, in both cases, the absence of P2X4 (P2X4KO) or the increase in surface P2X4 (P2X4KI) may lead to a decrease in the intracellular P2X4 pools. This hypothesis is however unlikely since, although P2X4mCherryIN are significantly more present at the surface than wild-type P2X4, the increase in surface trafficking is not associated with a depletion of intracellular P2X4mCherryIN pools as previously showed by intracellular staining of P2X4mCherryIN in various cell types including macrophages or neurons [48]. To go further in the understanding of the P2X4 pathophysiological cell-specific function and dynamic, conditional cell-specific P2X4KI or P2X4KO mouse lines expressing P2X4mCherryIN or invalidated for the P2X4 gene specifically in myeloid cells (macrophages and microglia) or in neurons should be useful to decipher time and cell-dependent P2X4 function as well as surface and intracellular contribution of P2X4 in ALS.

The polyclonal P2X4 antibody used previously to detect P2X4 expression in the spinal cord of SOD1 mice [30], was directed against the intracellular C-terminal domain of P2X4. The authors of this study showed later that this anti-P2X4 antibody also recognizes the misfolded SOD1 mutant protein [49] that shares sequence similarities with the P2X4 C-tail. These striking results stressed the fact that the previously observed increase in the expression of P2X4 may not be specific and may only reflect an increase in misfolded SOD1 proteins rather than an increase in P2X4 expression. We thus used novel specific monoclonal antibodies directed against extracellular domain of native mP2X4 [48, 60] or against the mCherry protein [48] to reexamine P2X4 expression in SOD1 mouse spinal cord. Immunostaining confirmed the low expression of P2X4 in spinal MNs of WT and P2X4KI mice observed using the d-tomato *p2rx4* reporter mice [61]. It is important to note that conditions leading to de novo expression of P2X4 in WT mice lead to de novo expression of non-internalized P2X4 in P2X4KI mice since P2X4mCherryIN is a modification of endogenous *p2rx4* gene remaining under the control of the native P2X4 gene promoter [48]. Our results revealed that P2X4 expression initially restricted to MNs (P75) in SOD1 or SOD1:P2X4KI increased during the symptomatic phase of ALS (P100) but only in microglia within the spinal cord likely due to a de novo expression of P2X4 in activated microglia as described in several pathological conditions [43, 61, 83]. qRT-PCR experiments confirmed that upregulation of microglia P2X4 at symptomatic phase of ALS is induced by IRF8-IRF5 transcriptional axis [76]. This apparent switch in P2X4 expression from neurons to microglia over the time might be may be related more to MN loss rather than the decrease in P2X4 expression by neurons.

The neuro-inflammatory marker changes observed in SOD1:P2X4KI or SOD1:P2X4KO for Iba1, P2Y12 and IL1-β suggest that P2X4 expression affects the microglial response in SOD1 mice in a stage dependent way. Although P2X4 is expressed in microglia and macrophages, these changes are likely related to microglia since CNS infiltration of peripheral myeloid cells have been suggested to be low in two transgenic SOD1 models [9]. Further, P2RY12 expression appears to be exclusively restricted to microglia and distinguishes microglia from infiltrating monocytes (for review [84]).Our data indicate that P2X4KO impacts differ depending on ALS stages. The level of Iba1 as well as P2Y12, two microglial homeostatic markers, are indeed reduced in SOD1:P2X4KO as compared to SOD1 mice at early stages. This might suggest that P2X4 deletion reduces the number of microglial cells or their proliferation. This point will remain to be further evaluated using 3D morphological approaches and transcriptomics. At later stages, we observed an enhanced IL1-β and P2Y12 expressions in SOD1:P2X4KO mice. Accordingly, recent data suggest that microglial P2Y12 is involved in NLRP3 inflammasome activation and therefore linked to IL1-β production, translating an overall pro-inflammatory event [84]. This might eventually explain the milder effect of P2X4KO towards the motor score as compared to SOD1:P2X4KI mice. On the other hand, as a homeostatic marker, enhanced P2Y12 expression might also relate to a beneficial anti-inflammatory outcome, providing that loss of P2Y12 expression in microglia has been reported in numerous neuropathologies related to neuroinflammation. This idea would fit with the reduction of TNFα observed in SOD1:P2X4KO mice. The relative contribution of P2Y12, IL1-β and TNFα changes in this animal models remain; therefore, difficult to interpret at this stage and would definitively deserve further evaluation.

P2X4 is highly expressed in macrophages [72, 85–87] that have recently been shown to be implicated in ALS pathogenesis [88–91]. Another major finding of the present study is that P2X4 surface density is significantly upregulated in SOD1 peripheral macrophages at early stages, long before the onset of ALS motor signs (P40-P75) and all along the progression of ALS (P100-P120). The significant rise of surface/total P2X4 ratio measured in SOD1 macrophages compared to WT mice is consistent with our data demonstrating that misfolded SOD1 proteins resembling to the C-terminal domain of P2X4 receptors [58] alter the constitutive internalization of P2X4, leading to increase P2X4 surface density in cell expressing this receptor. We observed a potentiation of ATP-induced P2X4 currents as well as an increase in P2X4 surface/total ratio in heterologous cells co-expressing mP2X4 receptors and different ALS human mutant SOD1 proteins (SOD1-G93A, G85R and G37R) compared to cells expressing WT human SOD1. These results reveal that the presence of mutant SOD1 proteins causes P2X4 surface upregulation as observed in vivo in SOD1 mice. In addition, our data demonstrated that misfolded mutant SOD1 proteins in ALS interfere with AP2-P2X4 interaction thereby altering P2X4 constitutive endocytosis and consequently leading to P2X4 surface upregulation. Co-immunoprecipitation revealed that misfolded SOD1 proteins interact specifically with AP2. Importantly, this interaction increases over the time of ALS progression in the spinal cord of SOD1 mice. Conversely, pull-down assay using as bait the C-terminal domain of P2X4 confirms that AP2 interacts with P2X4 C- tail [58, 92]. Moreover, the interaction between AP2 and P2X4 C-tail decreases over the time in SOD1 mice confirming that misfolded SOD1 proteins alter P2X4 endocytosis. Although we cannot completely rule out a possible participation of intracellular pools of P2X4, our data point out an important role of surface-expressed P2X4 in the pathogenesis of ALS.

Since P2X4 is expressed in human macrophages and monocytes [72], changes in P2X4 surface/total ratio from peripheral blood cells may represent an early marker of ALS. Moreover, our results show that the high P2X4 density observed in macrophages of P2X4KI mice phenocopy the increase observed in SOD1 macrophages. Peripheral macrophages in contact with axons of spinal MNs contribute directly to ALS pathogenesis and neurodegeneration [9] and have the capacity to modify the progressive microglia activation and proinflammatory responses. Since P2X4 is expressed in macrophages and MNs and increase in microglia during ALS, the constitutive higher surface P2X4 density in P2X4KI mice may promote distinct and specific effects in different cell types and modulate neurodegenerative processes in ALS. This may explain why SOD1:P2X4KI mice display delayed symptoms and increased survival although misfolded proteins increase surface P2X4 density in ALS.

## Conclusion

**Fig. 7:**
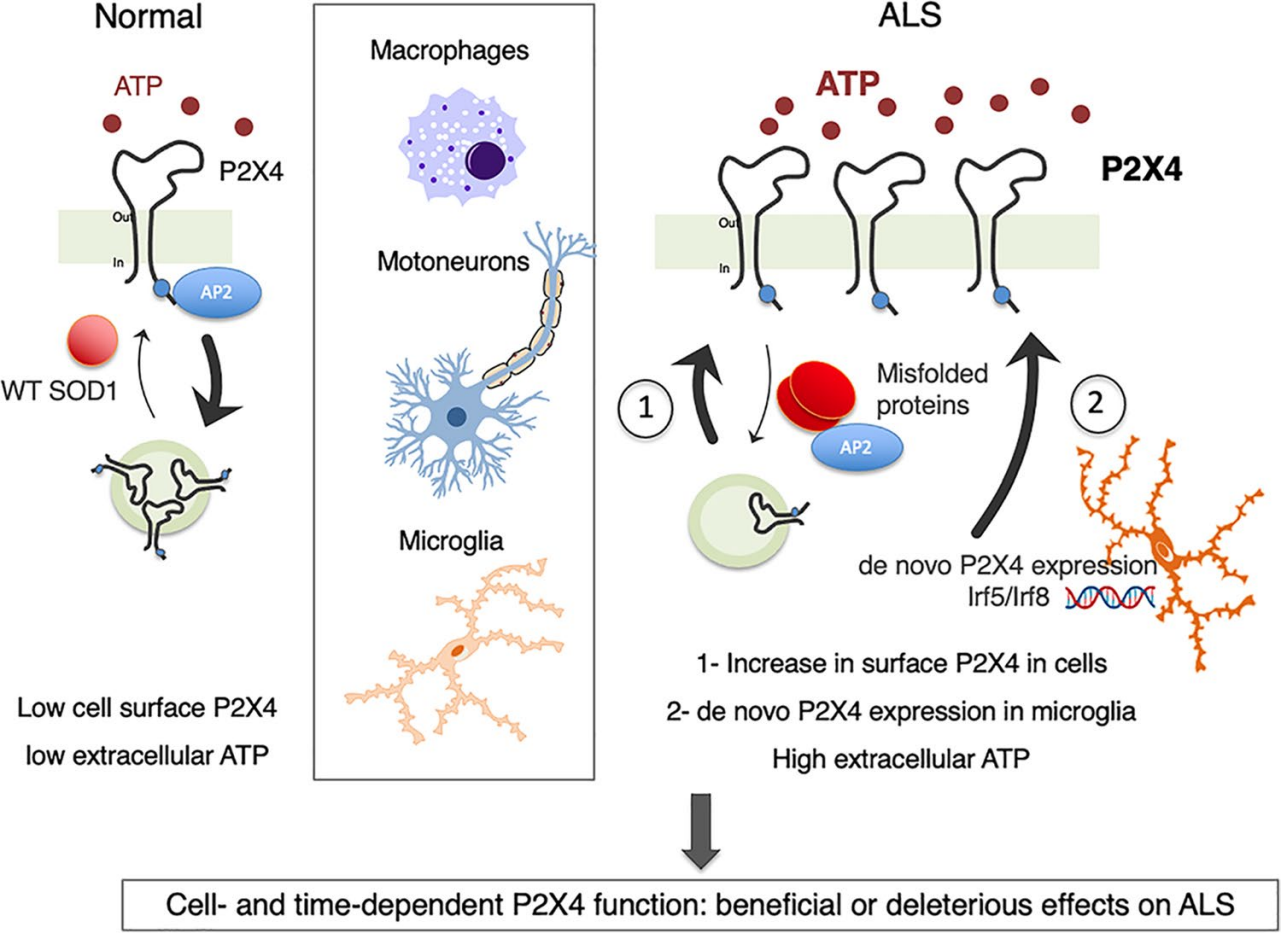
Proposed mechanism of surface P2X4 upregulation and consequences in ALS**.** In normal conditions P2X4 is constitutively endocytosed by the binding of AP2 on its C-terminus internalization domain resulting in a low surface expression restricted to MN within the spinal cord. During ALS progression, (1) misfolded mutant proteins like SOD1 or TDP-43 interfere with P2X4 internalization by competing for AP2 interaction leading to an increase in surface P2X4 density in cells expressing P2X4 such as MNs and macrophages at early stages. (2) At symptomatic stages de novo P2X4 expression in spinal reactive microglia further increase P2X4 signaling in microglia. Cell-specific and time-dependent activation of P2X4 is critical for beneficial or detrimental effects on ALS

The present work provides a better comprehension of P2X4 involvement and trafficking dynamics during the pathogenesis of ALS in the SOD1 mouse model. We demonstrated that misfolded SOD1 proteins alters P2X4 surface trafficking that contributes to ALS motor signs and survival of SOD1 mice. Cell-specific and time-dependent activation of P2X4 appears critical for beneficial or detrimental effects on ALS symptoms (Fig. 7). Future work should therefore determine whether manipulating P2X4 in a cell-specific manner might be a promising therapeutic strategy for fighting against ALS. Furthermore, P2X4 being expressed in macrophages but also in peripheral monocytes [93, 94], it will be of interest to investigate whether the upregulation of surface P2X4 herein observed in peripheral macrophages of SOD1 mice also occurred in monocytes of ALS patients. Detection of P2X4 from human peripheral cells may thus represent a unique and early biomarker to detect ALS before symptom occurrence.

## Supplementary Information

Below is the link to the electronic supplementary material.

Supplementary file1 (PDF 13186 KB)

## Data Availability

The datasets used and/or analyzed during the current study are available from the corresponding author on reasonable request.

## Abbreviations

AP2: Adaptor protein-2
AD: Alzheimer disease
ALS: Amyotrophic lateral sclerosis
FALS: Familial amyotrophic lateral sclerosis
FUS: Fused in sarcoma
GFAP: Glial fibrillary acidic protein
Iba1: Ionized calcium-binding adaptor molecule 1
IL: Interleukin
KO: Knock-out
mP2X4: Mouse P2X4
IRF5: Interferon regulatory factor 5
IRF8: Interferon regulatory factor 8
MS: Multiple sclerosis
NGS: Normal goat serum
PBS: Phosphate buffer saline
P2X4KI mice: Internalization-defective P2X4mCherryIN knock-in mice
PFA: Paraformaldehyde
RT-qPCR: Reverse transcriptase quantitative polymerase chain reaction
RFP: Red fluorescent protein
SALS: Sporadic amyotrophic lateral sclerosis
SDS-PAGE: Sodium dodecyl sulfate–polyacrylamide gel electrophoresis
SOD1: Superoxide dismutase-1
TBS: Tris Buffer Saline
TDP-43: TAR DNA-binding protein
TNFα: Tumor necrosis factor
WT: Wild type
