# Supplementary material for: Increased surface P2X4 receptors by mutant SOD1 proteins contribute to ALS pathogenesis in SOD1-G93A mice
Source: Cell Mol Life Sci. 2022 Jul 19;79(8):431. doi: 10.1007/s00018-022-04461-5 (PMC9296432; doi:10.1007/s00018-022-04461-5)
Supplement: Supplementary file 1 — Supplementary file1 (PDF 13186 KB) [file 18_2022_4461_MOESM1_ESM.pdf]

Supplementary Information

Cellular and Molecular Life Sciences

Increased surface P2X4 receptors by mutant SOD1 proteins contribute to ALS pathogenesis in SOD1-G93A mice

Eléonore Bertin, Audrey Martinez, Anne Fayoux, Kevin Carvalho, Sara Carracedo, Pierre-Olivier Fernagut, Friedrich Koch-Nolte, David Blum, Sandrine S. Bertrand and Eric Boué-Grabot*
*Corresponding author: email: eric.boue-grabot@u-bordeaux.fr

This file contains 9 supplementary Figures and legends and 1 supplementary Table S1.

Supplementary Figs and Legends

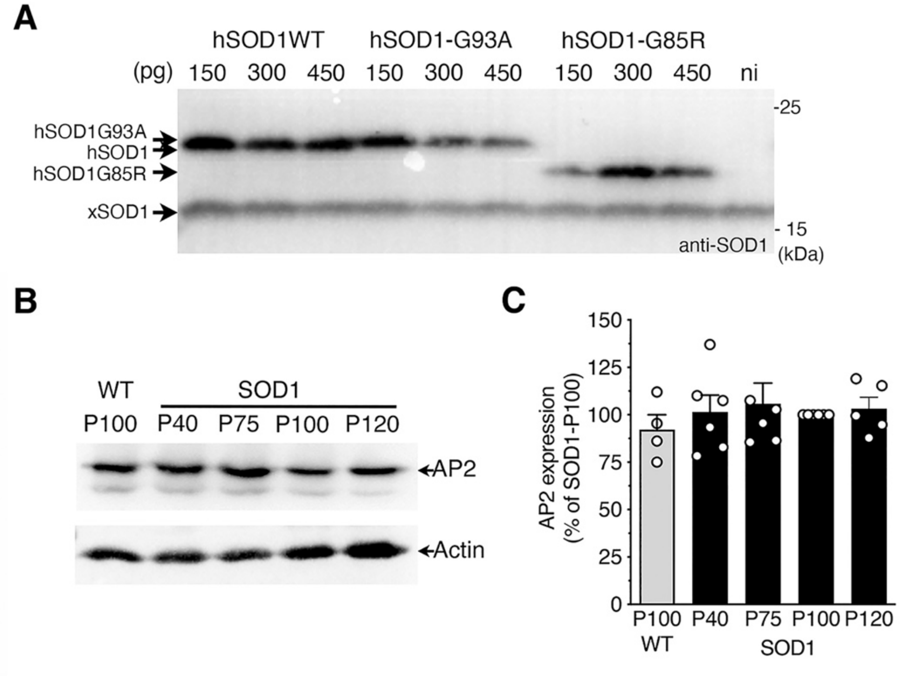

Fig. S1. A Western blotting of total proteins from *Xenopus* oocytes injected with different quantities of cDNA coding for hSOD1-WT, hSOD1-G93A or hSOD1-G85R and non-injected oocytes (ni) using anti-SOD1 antibodies to determine the quantities needed to obtain similar expression of the WT or mutated hSOD1 proteins in co-expression experiments with P2X4. Endogenous Xenopus SOD1 (xSOD1) was also detected. **B, C** Western blot analysis of spinal cord protein extracts from wild type (WT, P100) and SOD1 mice at different stages (P40 to P120) using anti-AP2 antibodies (AP50) and anti-actin as loading control showed that total AP2 expression was similar in WT and SOD1 mice over the time and progression of the disease (B). The expression of the SOD1 protein was normalized to the expression measured in P100 SOD1 mice in the different conditions tested (C).

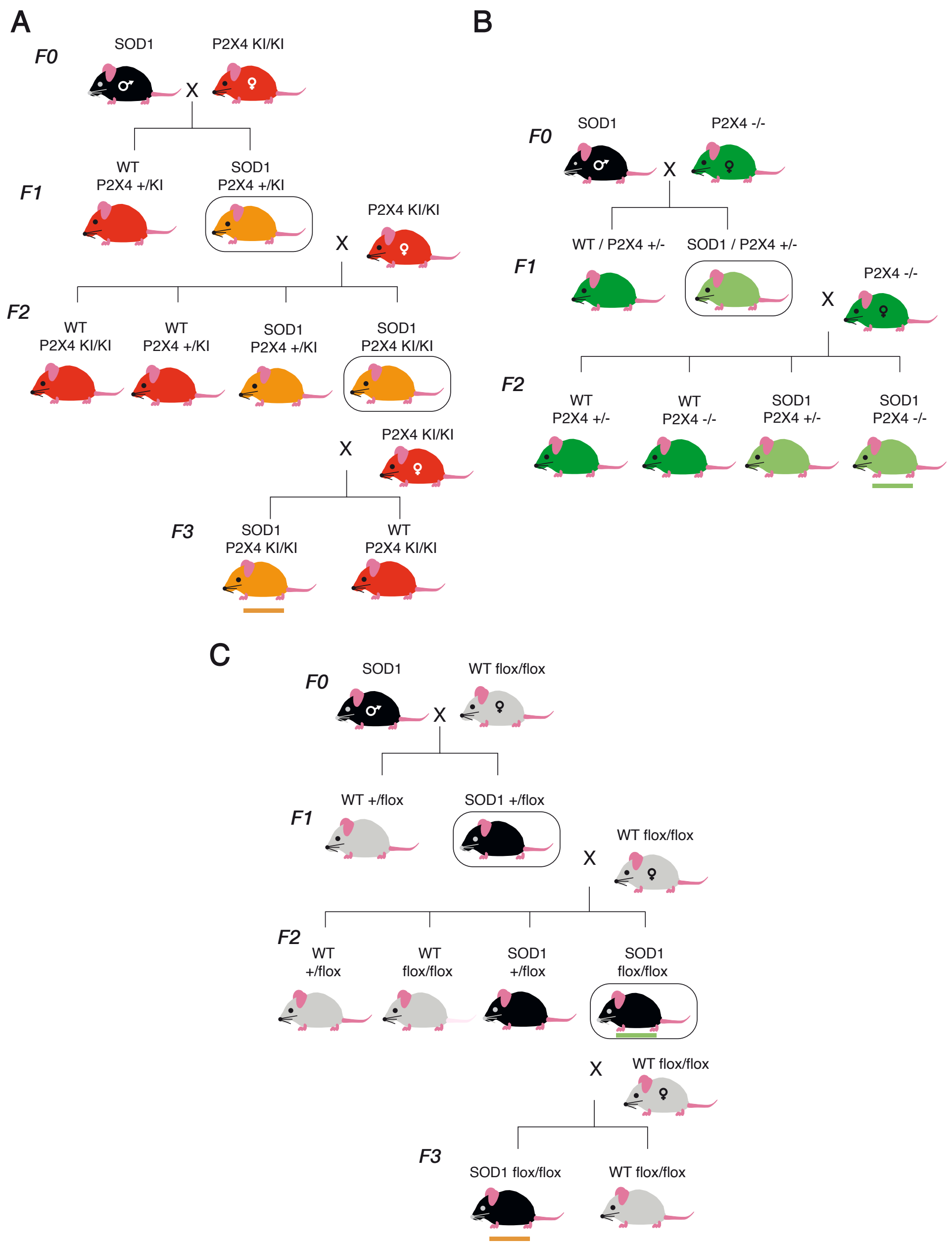

Fig. S2. Breeding scheme. **A** Breeding of SOD1 mice with constitutive P2X4KI mice to obtain third generation (F3) SOD1:P2X4KI (orange bar). P2X4KI mice were initially obtained by breeding flox/flox mice with CMV-Cre mice. **B** Breeding of SOD1 mice with constitutive P2X4KO mice to obtain SOD1:P2X4KO used in second generation (F2, green bar). **C** Breeding scheme of the SOD1:WT and control (WT:WT) animals used. WT floxed mice are flox/flox P2X4KI mice not crossed with CMV-Cre mice and expressing P2X4WT. SOD1 mice were mated with WT floxed mice (flox/flox) to obtain F2 (green bar) and F3 (orange bar) SOD1:WT mice presenting the same genetic background than F2 SOD1:P2X4KO and F3 SOD1:P2X4KI, respectively. WT: flox/flox were used as control mice (WT:WT).

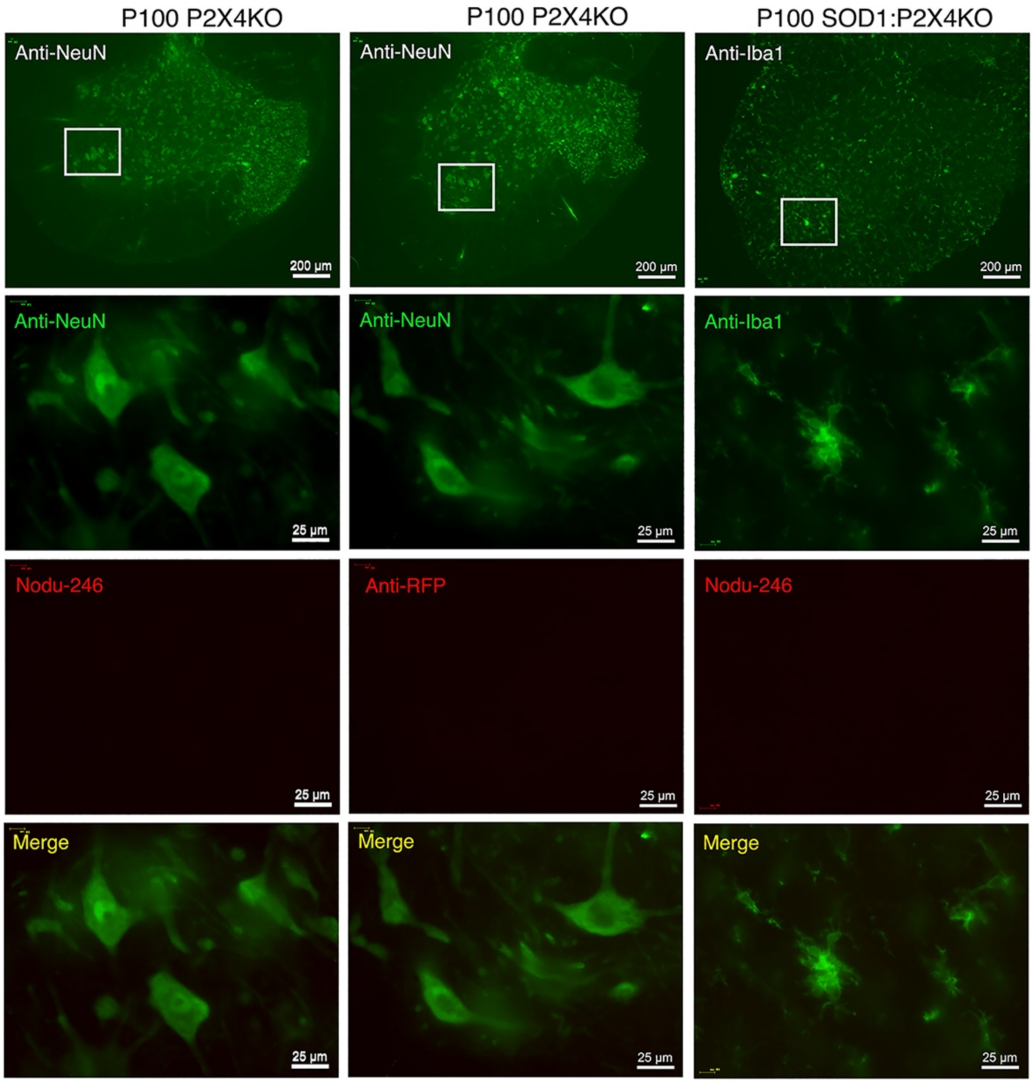

Fig. S3. No signal is detected using rat monoclonal anti-P2X4 (Nodu-246) or anti-RFP in spinal cords of P100 WT:P2X4KO (P2X4KO) or SOD1:P2X4KO mice showing that both antibodies recognized specifically P2X4 or P2X4mCherryIN.

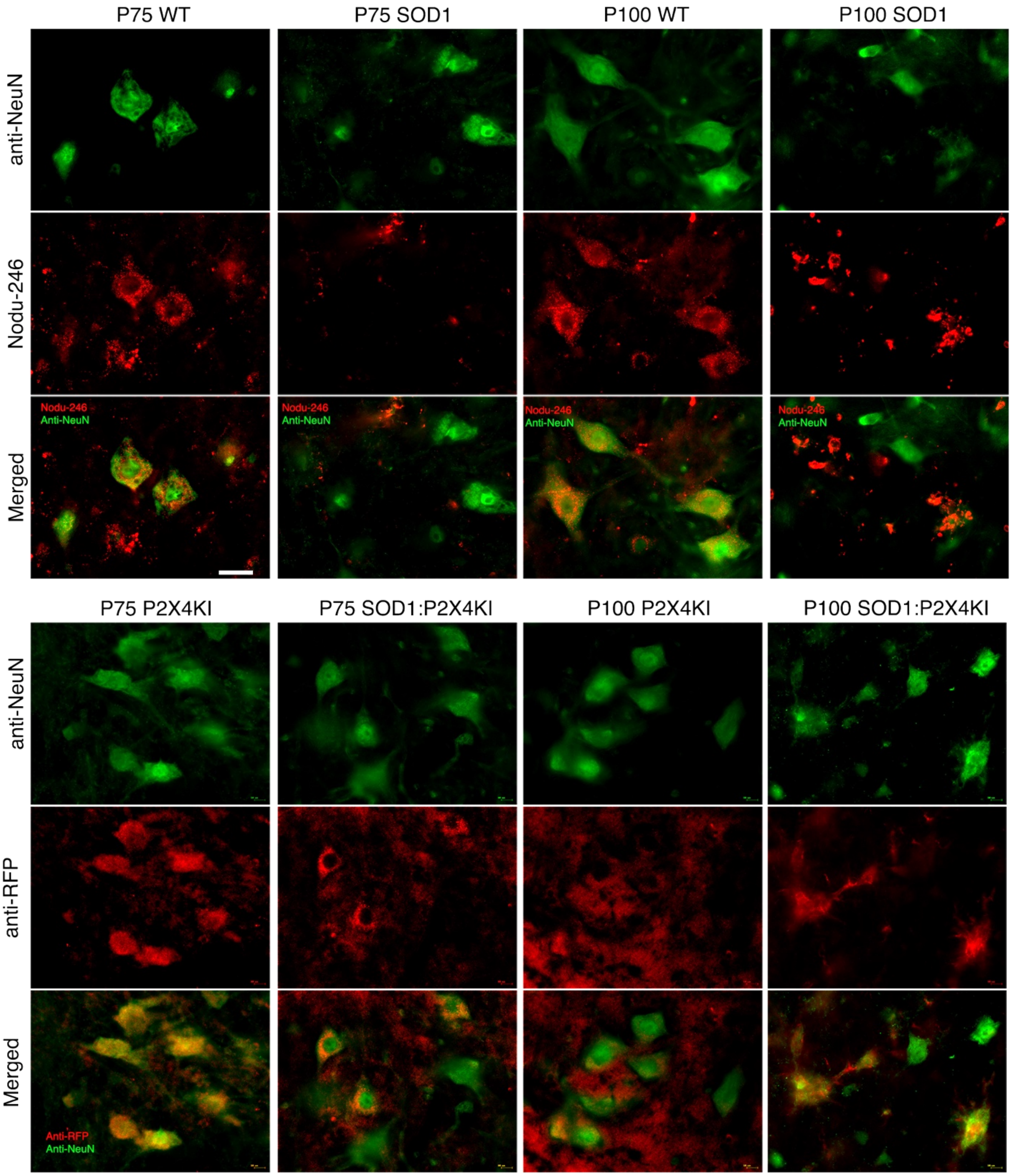

Fig. S4. Expression of P2X4 (red) in neurons (anti-NeuN green) in WT, SOD1:WT (SOD1), WT:P2X4KI (P2X4KI) and SOD1:P2X4KI mice at pre- (P75) and symptomatic (P100) phases of ALS revealed with a rat monoclonal P2X4 antibodies (Nodu-246) and expression of P2X4KI revealed with anti-RFP in P2X4KI or SOD1:P2X4KI mice. Individual and merged staining of overlays showed in Fig. 4A. Scale bar 25 μm.

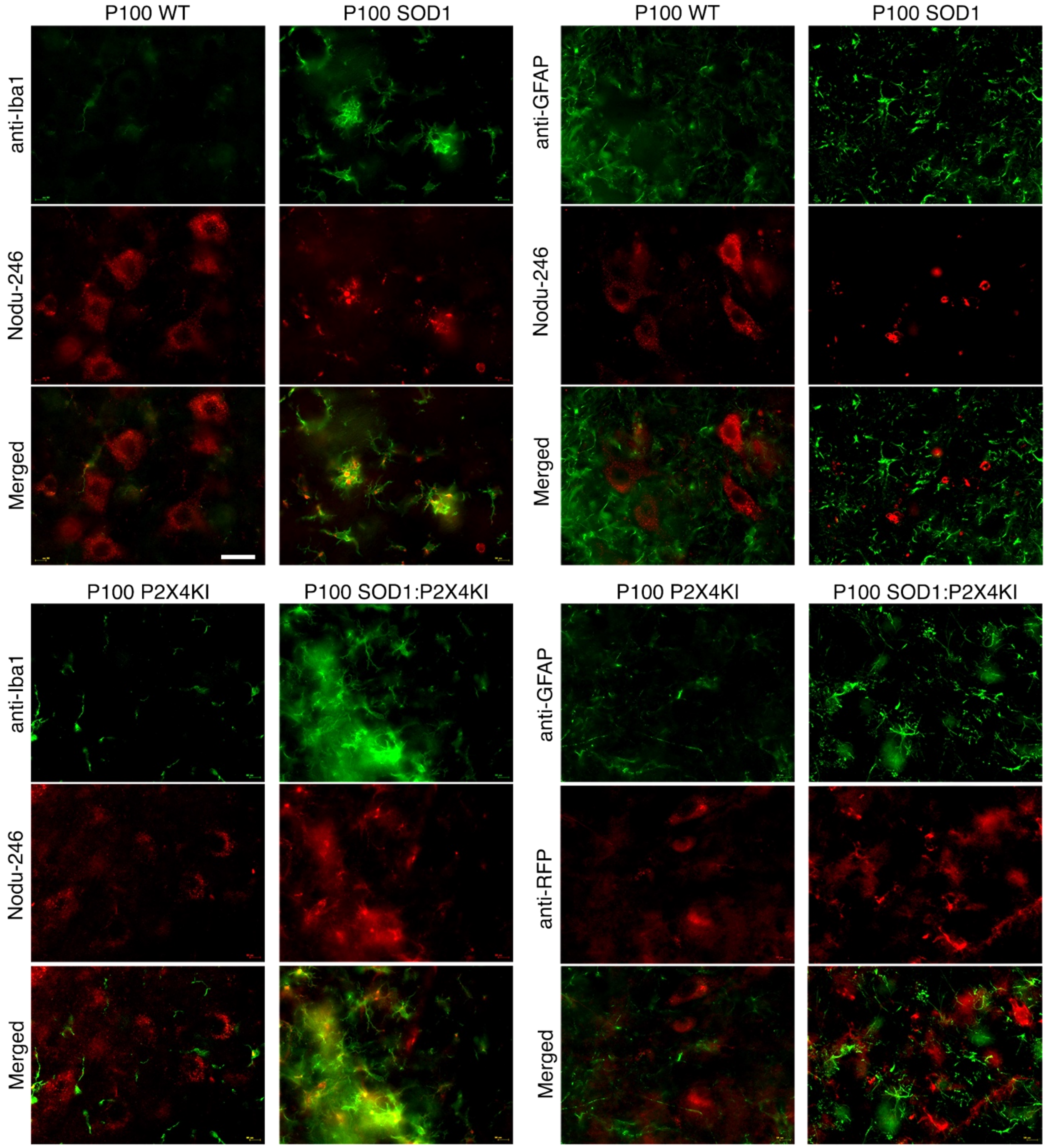

Fig. S5. Expression of P2X4 (red) in microglia (anti-Iba1, green) or astrocytes (anti-GFAP, green) (anti-NeuN green) in P100 WT, SOD1:WT (SOD1), WT:P2X4KI (P2X4KI) and SOD1:P2X4KI mice revealed with a rat monoclonal P2X4 antibodies (Nodu-246) and or anti-RFP. Individual and merged staining of overlays showed in Fig. 4B, C. Scale bar 25 μm.

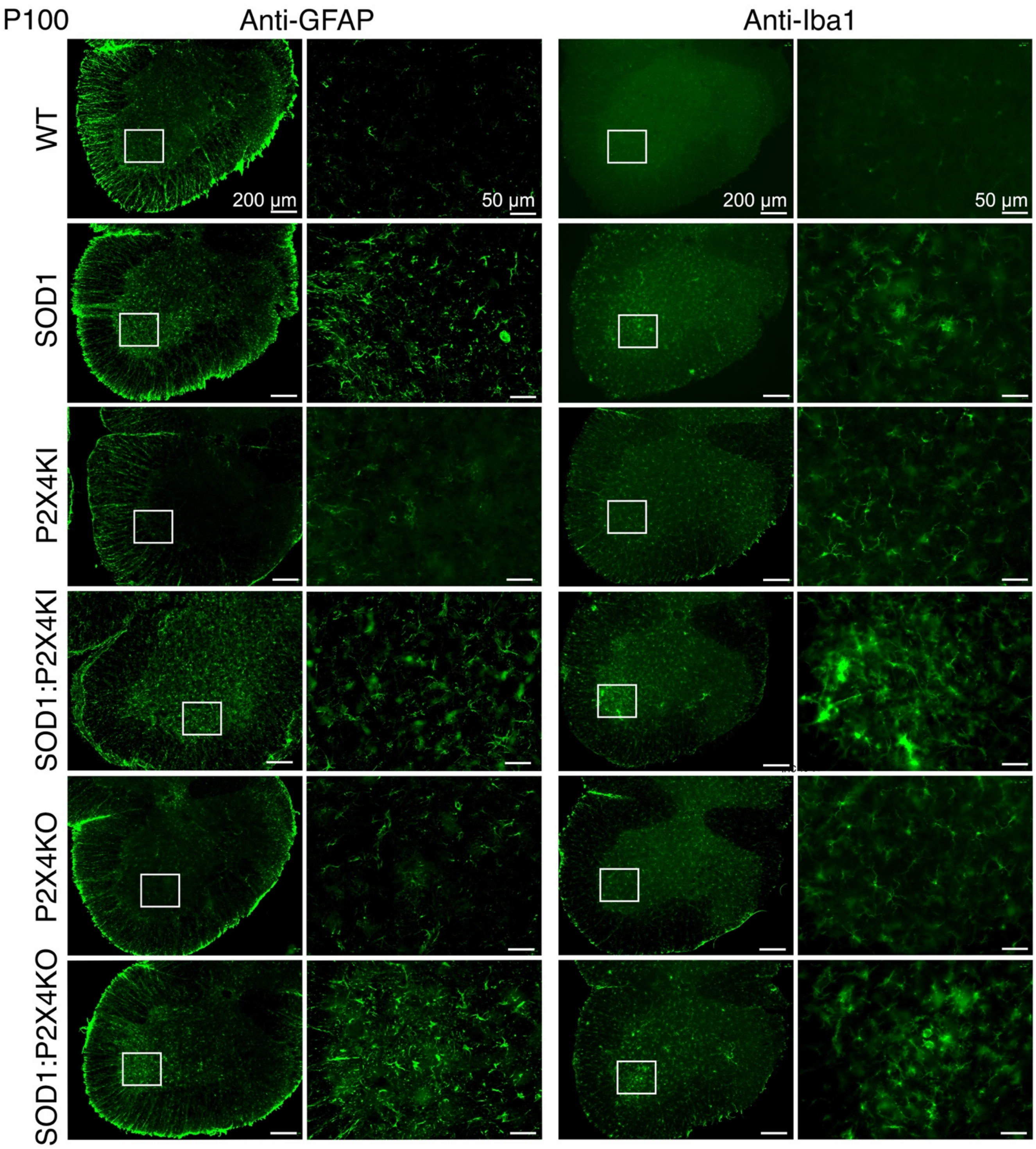

Fig. S6. GFAP (left) and Iba1(right) immunodetection in the spinal cord of the different mouse lines used in this study at P100. GFAP positive astrocytes are localized in the white matter in control mice and have invaded the grey matter in P100 SOD1 mice. Similar changes were observed in SOD1:P2X4KI and SOD1:P2X4KO animals when compared to control P2X4KI or P2X4KO mice. Iba1 positive microglia labeling begins to appear in P75 SOD1 mice and increases drastically in P100 SOD1 mice.

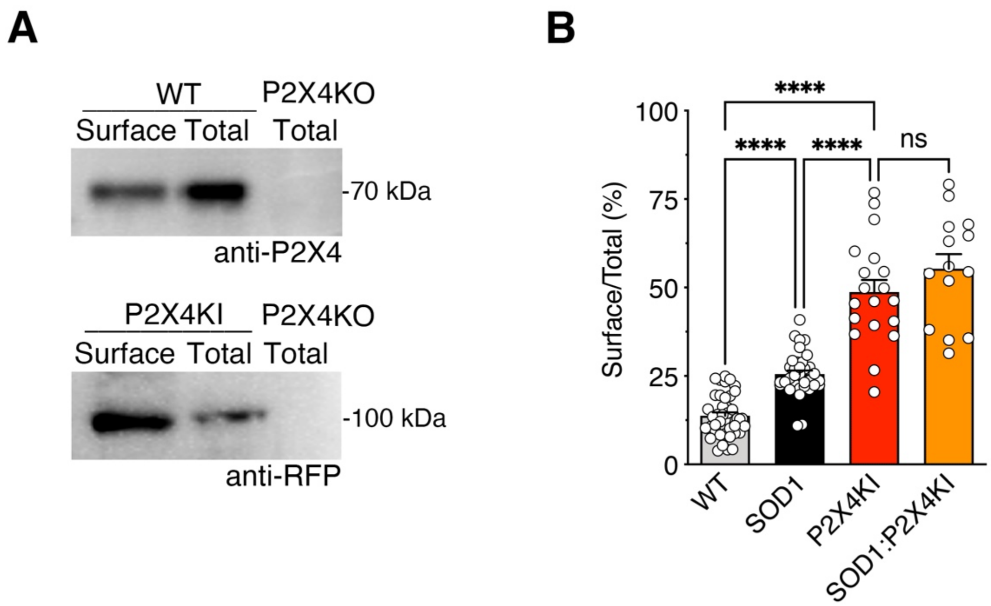

Fig. S7. Surface/total P2X4 in macrophages of the different mouse lines. **A** Control western blotting of total and biotinylated surface proteins from peritoneal macrophages isolated from WT:WT (WT), WT:P2X4KI (P2X4KI) or WT:P2X4KO (P2X4KO) mice (P100) revealed using anti-P2X4 or anti-RFP antibodies as indicated on the gels. **B** Surface/total ratio measured from macrophages of the different mouse lines collected at different time points (P40, P75 and P100) presented individually in Fig. 5 are here presented pooled for each mouse type. Results confirm that i) P2X4 surface density is significantly higher in SOD1 mice compared to WT mice independently from the disease stage; ii) the increase in the number of surface P2X4 is higher in WT:P2X4KI (P2X4KI) mice than in WT ones as previously shown [48]; iii) this increase remained unchanged in SOD1:P2X4KI as compared to WT:P2X4KI mice. **** p<0.0001, one-way ANOVA).

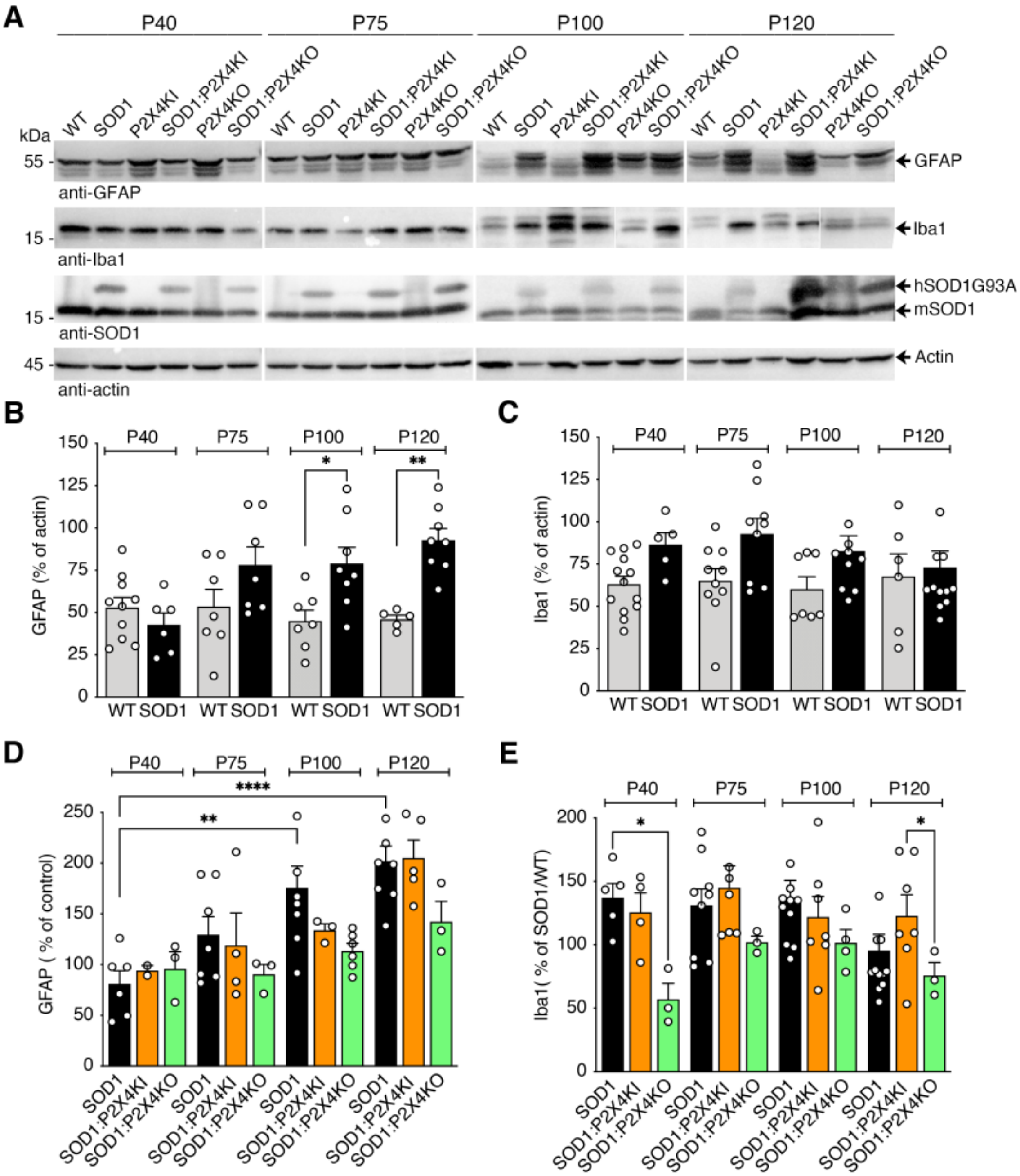

Fig. S8. Expression of GFAP, Iba1, SOD1 and β-actin of the different mouse lines over time. **A** Detection of GFAP, Iba1, SOD1 and β-actin by western blotting of total proteins extracted from spinal cords of WT and SOD1:WT (SOD1), WT:P2X4KI (P2X4KI), SOD1:P2X4KI as well as WT:P2X4KO (P2X4KO) and SOD1:P2X4KO at indicated stages. The anti-SOD1 antibody revealed 2 bands of different size corresponding to the endogenous mouse (m)SOD1 and the human (h)SOD1-G93A proteins confirming the genotype of the mice (WT or SOD1). Anti-Actin was used as a control loading and to normalize measurements. **B-C** Graph bars showing the expression of GFAP and IBA1 detection for WT and SOD1 mice over the time. **D-E** Changes of GFAP and Iba1 for SOD1, SOD1:P2X4KI, and SOD1:P2X4KO mice compared to the level of their respective control mice (WT, P2X4KI and P2X4KO), respectively. * p < 0.05, ** p<0.01, *** p<0.001 two-way ANOVA.

A

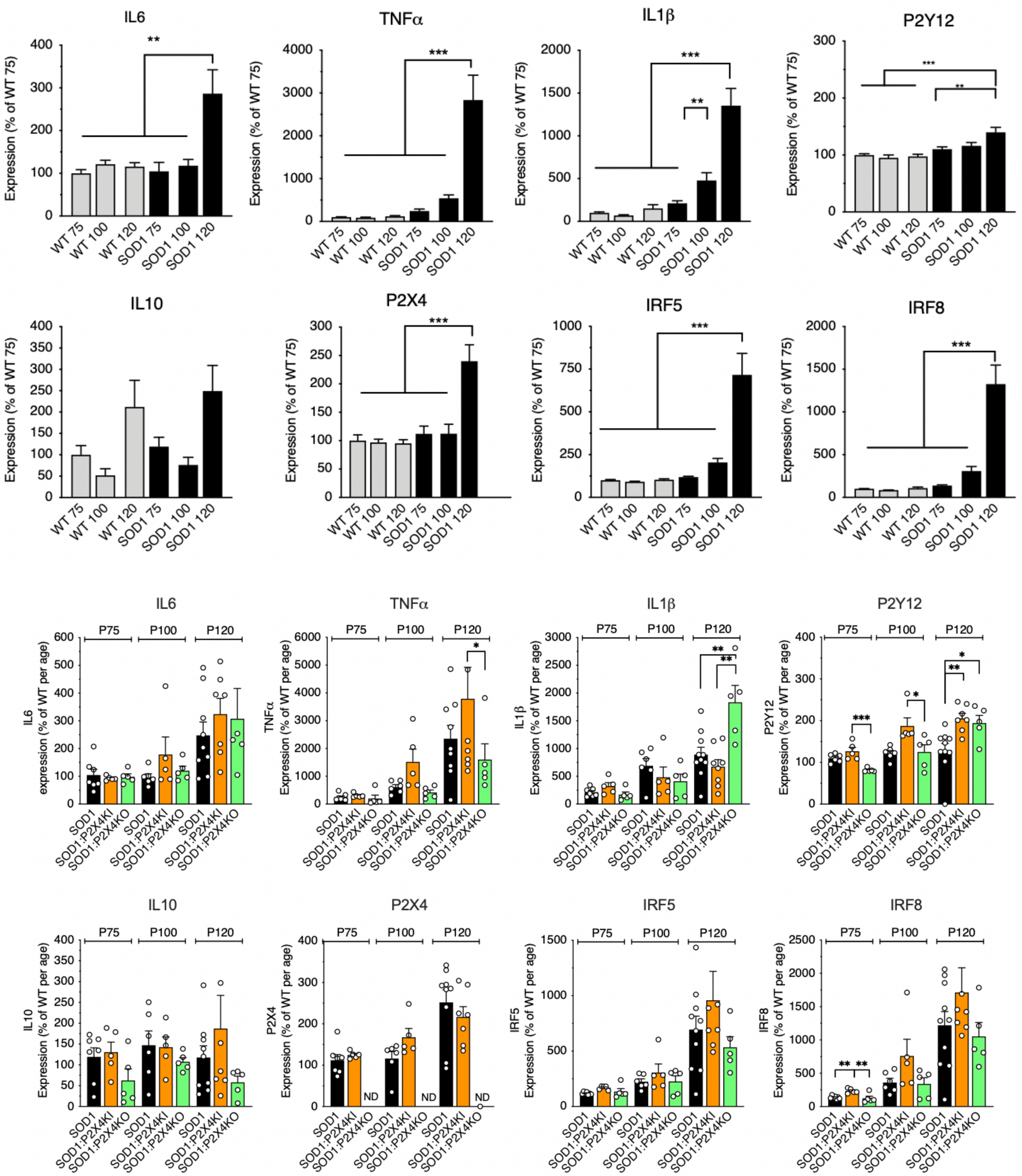

Fig. S9. Gene expression levels of classical inflammatory markers involved in ALS. **A** (IL6, TNFα, IL1β, IL10), homeostatic microglial gene (P2Y12), P2X4 gene and two transcriptional factors of P2X4 (IRF5, IRF8) evaluated using qPCR in the spinal cord of WT and SOD1:WT (SOD1) mice. **B** Changes of expression in SOD1, SOD1:P2X4KI, and SOD1:P2X4KO mice compared to the level of their control mice at the same age (WT, P2X4KI and P2X4KO) respectively. * p < 0.05, ** p<0.01, *** p<0.001 two-way ANOVA.

Table S1. List of reagents used in this study

Reagent/Resource	Reference or Source	Identifier
**Organisms**		
Floxed knockin P2X4mCherryIN mice	Bertin et al. 2021	N/A
CMVCre+-P2X4mCherryIN mice (P2X4KI)	Bertin et al. 2021	N/A
B6.C-Tg(CMV-cre)1Cgn/J	Jackson Laboratory	Jax #006054
P2X4KO mice	Sim et al, 2006 A gift of F. Rassendren IGF, Montpellier, France	N/A
B6SJL SOD1-G93A	Jackson Laboratory	Cat#002726
C57BL/6J mice	Jackson Laboratory	Jax #000664
Xenopus laevis	European Xenopus Resource Centre (EXRC), Porthmouth UK	N/A
**Primary Antibodies**		
Nodu-A246 rat monoclonal against native extracellular domain of mouse P2X4 antibody	Bergmann et al 2019<br>Bertin et al. 2021	N/A
Rabbit anti-P2X4	Alomone	Cat#APR-002
Rabbit anti-RFP	MBL	Cat#PM005
Chicken anti-GFAP	Abcam	Cat#ab4674
Rabbit anti-Iba1	Wako	Cat#19-197441
Mouse anti-NeuN	Merk	Cat#MAB377
Mouse anti-actin	Sigma Aldirch	Cat#A5441
Goat anti-SOD1	R&D Systems	Cat#AF3787
Anti-AP2	BD Bioscience	Cat#61351
**Fluorescent secondary antibodies**		
Anti-rat AlexaFluor568™	Life technologies	Cat#A-11077
Anti-rabbit AlexaFluor568™	Life technologies	Cat#A11011/<br>Cat#A10042
Anti-mouse AlexaFluor488™	Life technologies	Cat#A11001
Anti-chicken AlexaFluor488™	Life technologies	Cat#A11001
Anti-rabbit AlexaFluor488™	Life technologies	Cat#A32731
**HRP-conjugated secondary antibodies**		
Anti-rabbit HRP-conjugated secondary antibodies	Jackson Immunoresearch	Cat#111035144

Anti-mouse HRP-conjugated secondary antibodies	Jackson Immunoresearch	Cat#115035062
Anti-goat HRP-conjugated secondary antibodies	Santa Cruz	Cat#SC2020
Anti-chicken HRP-conjugated secondary antibodies	Jackson Immunoresearch	Cat#703-035-155
**Chemicals**		
Formalin solution neutral buffered 10%, 4% PFA	Sigma-Aldrich	Cat#HT501128
EZ-LINK SULFO-NHS-SS-BIOTIN	Fisher	Cat#11811205
HIGH CAP NEUTRAVIDIN AGA RESIN	Fisher	Cat#10742135
X10 PAGERULER PLUS PRES PROT LAD	Fisher	Cat#11852124
Clarity ECL substrate	Biorad	Cat#1705061
Pierce™ BCA Protein Assay Kit	Fisher	Cat#23225
Vectashield with DAPI	Vectors Laboratories	H-1200
Dynabeads™ Antibody Coupling kit	Invitrogen (ThermoFicher Scientific)	Cat#14321D
Kit Pierce™Pull-Down Biotinylated Protein:Protein Interaction	Pierce	Cat#2115
RNeasy Lipid Tissue Mini Kit	Qiagen	Cat#74804
High-Capacity cDNA reverse transcription kit with RNase inhibitor	Applied Biosystems	Cat#4374966
TaqMan™ Gene Expression Master Mix	Applied Biosystems	Cat#4305719
**Recombinant DNA**		
mP2X4WT-pcDNA3	Toulmé et al. 2006	N/A
mP2X4mCherryIN-pCDNA3	Bertin et al. 2021	N/A
hSOD1WT-pcDNA3	This manuscrit	N/A
hSOD1G93A-pcDNA3	This manuscrit	N/A
hSOD1G85R-pcDNA3	This manuscrit	N/A
hSOD1G37R-pcDNA3	This manuscrit	N/A
hTDP-43	Addgene	#28206
**Software**		
ImageJ /Fiji	National Institutes of Health, USA	https://imagej.nih.gov/ij/
Prism	Graphpad	www.graphpad.com
Axograph X	Axograph	https://axograph.com
**qPCR probe**		
Taqman probe IL10	Applied Biosystems	Mm01288386_m1

Taqman probe IL1β	Applied Biosystems	Mm00434228_m1
Taqman probe IL6	Applied Biosystems	Mm00446190_m1
Taqman probe IRF5	Applied Biosystems	Mm00496477_m1
Taqman probe IRF8	Applied Biosystems	Mm00492567_m1
Taqman probe P2X4	Applied Biosystems	Mm00501787_m1
Taqman probe P2Y12	Applied Biosystems	Mm00446026_m1
Taqman probe PPIA	Applied Biosystems	Mm02342430_g1
Taqman probe TNFα	Applied Biosystems	Mm00443258_m1
